# Molecular Insight into the Inhibition of *Pseudomonas aeruginosa* Quorum Sensing by 5‑Bromoindole-3-carboxaldehyde and Curcumin: A Combined In Vitro Study, Molecular Dynamics, and Docking Studies

**DOI:** 10.1021/acsomega.6c02161

**Published:** 2026-07-06

**Authors:** Gabriel Martínez González, José Marcos Falcón-González, Rodolfo García Contreras, Juan Andres Alvarado-Salazar, Israel García Valdés, Nilda García Valdés, Denisse Alejandra Lugo Gutiérrez, Ingrid Lizeth Bustamante Martínez, Alonso Ruben Tescucano Alonso, Alejandra Ramirez-Villalva, Jorge A. Almeida

**Affiliations:** † Instituto de Investigación y Estudios en Salud (INIES), Universidad de Ixtlahuaca CUI, Ixtlahuaca Jiquipilco Manzana 067, San Pedro 50740, San Pedro la Cabecera, Mexico; ‡ Unidad Profesional Interdisciplinaria de Ingeniería Campus Guanajuato, 27740 Instituto Politecnico Nacional, Avenida Mineral de Valenciana 200 Col. Fraccionamiento Industrial Puerto Interior, 36275 Silao de la Victoria, Guanajuato, Mexico; § Departamento de Microbiología y Parasitología, Facultad de Medicina, 7180 Universidad Nacional Autonoma de Mexico, Circuito Interior s/n, Avenida Universidad 3000, Ciudad Universitaria, Coyoacán 04510, Mexico; ∥ Carrera de Química Farmacéutico Biológica, Área Farmacéutica, Facultad de Estudios Superiores ″Zaragoza″, Universidad Nacional Autonoma de Mexico, Batalla 5 de Mayo S/N, Ejército de Oriente Zona Peñón, Iztapalapa 09230, Mexico; ⊥ Academia de Farmacología y Toxicología, Facultad de Estudios Superiores ″Zaragoza″, Universidad Nacional Autonoma de Mexico, Batalla 5 de Mayo S/N, Ejército de Oriente Zona Peñón, Iztapalapa 09230, Mexico; # Laboratorio de Biofísica y Biocatálisis, Sección de Estudios de Posgrado e Investigación, Escuela Superior de Medicina, Instituto Politecnico Nacional, Salvador Díaz Mirón esq. Plan de San Luis S/N, Miguel Hidalgo, Casco de Santo Tomas, Miguel Hidalgo, Mexico 11340, Mexico; ¶ Licenciatura en Química Farmacéutica Biológica, Universidad de Ixtlahuaca, CUI, Ixtlahuaca Jiquipilco Manzana 067, San Pedro 50740, San Pedro la Cabecera, Mexico; ∇ Facultad de Química, Universidad Autonoma del Estado de Mexico, P.° Colón S/N, Residencial Colón y Col Ciprés, 50120 Toluca de Lerdo, Mexico; ○ Escuela Profesional de Medicina, Universidad de Ixtlahuaca CUI, Ixtlahuaca Jiquipilco Manzana 067, San Pedro 50740, San Pedro la Cabecera, Mexico

## Abstract

Curcumin and 5-bromoindole-3-carboxaldehyde were tested for their ability to inhibit quorum sensing-dependent virulence factors in *Pseudomonas aeruginosa* strains Pa01, Pa14, and ATCC 27853. The compounds were evaluated at curcumin (1 and 5 μg/mL) and 5-bromoindole-3-carboxaldehyde (30, 50, and 100 μg/mL). Virulence factors—exoprotease, elastase, pyocyanin, and alginate—were measured. Chemoinformatics properties were analyzed using Molinspiration, Molsoft, Osiris Property Explorer, pkCSM, and SwissADME. Results showed 5-bromoindole-3-carboxaldehyde significantly reduced pyocyanin in Pa01 (92.5% at 100 μg/mL), elastase (79.85% at 100 μg/mL), exoprotease (92.8% at 50 μg/mL), and alginate in ATCC 27853 (76.16% at 100 μg/mL). Curcumin effectively inhibited pyocyanin in ATCC 27853 (100% at 5 μg/mL), elastase in Pa14 (56.19% at 5 μg/mL), exoprotease in Pa01 (87.04% at 5 μg/mL), and alginate (48.26% at 5 μg/mL). Both compounds demonstrated strong inhibition of virulence factors, suggesting their potential as antivirulence agents against *P. aeruginosa*, a highly resistant bacterium prevalent in nosocomial infections.

## Graphical abstract

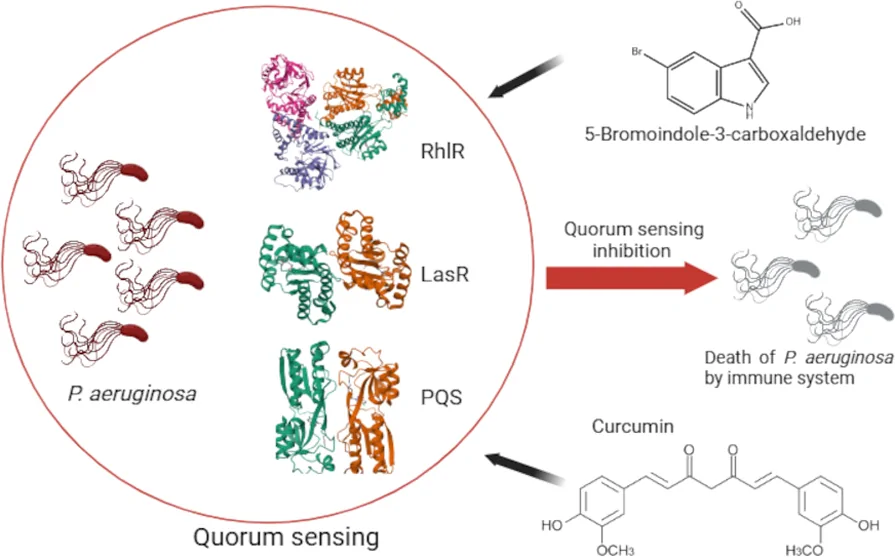

## Introduction

*Pseudomonas aeruginosa* is a Gram-negative aerobic rod-shaped bacterium that can be isolated from most environments, including soil, plants, and mammal tissue.^1^ It has become an important cause of infection in humans and can be associated with significant morbidity and mortality.^2^ It is a common cause of nosocomial infections, particularly pneumonia and infection in immunocompromised hosts and in those with structural lung disease such as cystic fibrosis.^3,4^

The World Health Organization (WHO) listed *P. aeruginosa* as one of three bacterial species in which there is a critical need for the development of new antibiotics to treat infections.^5^ Importantly, *P. aeruginosa* is one of the MDR ESKAPE pathogens.^6^

*P. aeruginosa* virulence regulation uses both chemical factors such as quorum sensing (QS) and mechanical cues such as surface association to modulate a large suite of virulence factors.^7^ An essential function for QS in *P. aeruginosa* is to regulate the production of multiple virulence factors, such as extracellular proteases, iron chelators, efflux pump expression, biofilm development, swarming motility, phospholipases C, alginate, DNase, and the response to host immunity.^8,9^

Resistance of *P. aeruginosa* to antibiotics is a major problem. Targeting virulence factors is an alternative option to avoid the emergence of resistance to antibiotics and treat infections that this pathogen causes.^10^ The QS system is an attractive target for antibacterial therapy.^11^ Inhibition of this system may cause the attenuation of virulence and protection against infection. In fact, an anti-QS approach has already shown promise in the battle against *P. aeruginosa* infections.^12^

The use of QS inhibitors would be of particular interest in inhibiting bacterial pathogenicity and infections.^13^ Turmeric (*Curcuma longa*) has traditionally been used as an anti-inflammatory, antimicrobial, antifungal, antioxidant, anticancer, anti-inflammatory, antiobesity, and antidepressant agent,^14^ but reports on its anti-QS properties are scarce.^15^ Several studies reported that curcumin inhibits bacterial QS systems/biofilm formation and prevents bacterial adhesion to host receptors in various species, including *Staphylococcus aureus*, *Enterococcus faecalis*, *Escherichia coli*, *Streptococcus mutans*, *Listeria monocytogenes*, *Helicobacter pylori*, *P. aeruginosa*, *Serratia marcescens*, *Aeromonas hydrophila*, and *Acinetobacter baumannii*.^16^

Another molecule whose reports indicate antibacterial activity is indole, which, although it uses bacteria as signaling, has shown important antibiofilm activity.^17^ Functional alteration of indole carboxaldehydes may increase their effectiveness as quorum-sensing inhibitors (QSIs). The bromination of carboxaldehydes represents an increase in their activity.^18^

Given the background of the antiquorum-sensing activity of molecules such as curcumin and carboxaldehydes, the objective of this work is to evaluate in three strains of *P. aeruginosa* (Pa01, Pa14, and ATCC 27853) the activity of curcumin and 5-bromoindole-3-carboxaldehyde on quorum-sensing-dependent virulence factors, such as alginate, pyocyanin, elastase, and protease.

The proposal of new antibacterial treatments often requires the use of computer software that allows the calculation of molecular properties and the prediction of bioactivity, with which the cheminformatics properties previously obtained can be correlated with the viability of the new treatment proposal. Chemoinformatic properties have advantages such as predicting the site of action, probable route of administration, physicochemical, pharmacokinetic, and pharmacodynamic behavior, and possible toxic effects. The use of simulation techniques and online servers allows us to know the behavior of a compound to propose an effective and safe therapy.

## Methods and Materials

### Preparation of Strains and Minimum Inhibitory Concentration

To carry out the tests, 3 different strains of *P. aeruginosa* were used: Pa01, Pa14 and ATCC 27853, all belonging to the microbiology laboratory of the University of Ixtlahuaca CUI. To activate these strains, they were reseeded in boxes of LB medium with 1.5% agar for at least 20 h at 37 °C.

The minimum inhibitory concentration (MIC) was developed using the microdilution method in a 96-well plate (Nunc), according to Table 10 of the Clinical and Laboratory Standards Institute (CLSI).^19,20^ To evaluate the minimum inhibitory concentration of each molecule, curcumin at concentrations of 1000, 500, 250, 125, 62.5, 31.25, and 15.62 μg/mL and 5-bromoindole-3-carboxaldehyde at concentrations of 1000, 500, 250, 125, and 62.5 μg/mL dissolved in DMSO were tested against strains Pa01, Pa14, and ATCC 27853. The inocula were adjusted to achieve 10^8^ CFU/mL of microorganisms. Subsequently, 10 μL of the corresponding inocula was added to the wells with Mueller Hinton Broth (MHB), obtaining a final concentration of 10^5^ CFU/mL.^21^ The negative controls were MHB without microorganisms, MHB with the *P. aeruginosa* strains, and MHB with DMSO and *P. aeruginosa* strains to confirm that it did not interfere with growth. The microplates were incubated at 35 ± 2 °C for 20 h. The analysis of each molecule was performed in triplicate.

### Preparation of the Strains and Addition of Evaluated Molecules

To activate these strains, Pa01, Pa14, and ATCC 27853, they were reseeded in boxes of LB medium with 1.5% agar for at least 20 h at 37 °C. Subsequently, ON (overnight) cultures of each strain were inoculated in 50 mL flasks with 25 mL of LB medium and cultivated for 18 h at 37 °C. After the elapsed time, the growth of the cultures was measured at OD of 600 nm in a 1:10 ratio (dilution in LB).

Subsequently, new cultures of the strains were inoculated from the ON cultures with an OD of 600 nm. The inoculums of each strain of interest were added to 3 series of flasks (each with 8 flasks with 25 mL of LB medium) since three different strains were evaluated; different concentrations of each molecule were added to each series: for curcumin (Sigma-Aldrich), they evaluated concentrations of 1 μg/mL and 5 μg/mL, and for 5-bromoindole-3-carboxaldehyde (Sigma-Aldrich), 30, 50, and 100 μg/mL; both molecules were dissolved in DMSO previously filtered with Acrodisc filters (0.22 μm). They were incubated at 37 °C with constant shaking for 24 h. To maintain control, a culture that did not have any of the added molecules was used. After 24 h, bacterial growth was measured as optical density at 600 nm (OD_600_) in both control and treated cultures. To account for potential variations in bacterial growth, all absorbance values obtained from the virulence factor assays (pyocyanin, elastase, protease, and alginate) were normalized to the corresponding OD_600_ for each culture. No significant differences in growth were observed between the treated and control groups under the experimental conditions. Since the molecules were dissolved in DMSO, appropriate solvent controls were included.

### Determination of Pyocyanin

From the mother flasks with ON, 1000 μL of each culture was taken. They were subsequently centrifuged at 13,000 rpm for 1.5 min. An 800 μL portion of the supernatant was taken. Adding 420 μL of chloroform, it was vortexed for 1 min at speed 10 and centrifuged at 13,000 rpm for 5 min. We transferred 300 μL of the bottom phase together with 800 μL of 0.1 N HCl. It was shaken for 1 min at vortex speed 10 and centrifuged at 13,000 rpm for 5 min. It was determined quantitatively by absorbance, measured in a quartz cell, at 520 nm.^22^

### Protease Determination

A 1000 μL portion was taken from the stock flasks with ON. The samples were centrifuged at 13,000 rpm for 2 min. An 875 μL portion of buffer with protease reagent and 125 μL of culture supernatant were used. It was vortexed for 1 min. Samples were incubated at 37 °C at 200 rpm for 1 h. At the end of the incubation time, aliquots were taken and placed in 1% HN0_3_. Shaking with the help of a vortex for 1 min at speed 10, we then centrifuged at 13,000 rpm for 2 min. The supernatant was placed in the presence of 0.5% Na0H. We stirred lightly with the help of the vortex. Transferring into quartz cells, the samples were read at 595 nm.^23,24^

### Determination of Alginate

A 1000 μL portion was taken from the stock flasks. A 1 ml portion of the cell culture from the cultures was centrifuged and subsequently heated at 80 °C for 30 min. The resulting supernatant was centrifuged for 30 min at 13,000 rpm, the pellet was removed, and the alginate was precipitated with ice-cold 99% ethanol. The precipitated alginate was collected and dissolved in 1 mL of 0.9% sterile saline. A mixture of 118 μL of sample plus 1 mL of H_3_BO_3_/H_2_SO_4_ and 34 μL of carbazole was made. This mixture was heated for 30 min at 55 °C, and the absorbance (OD 530) was measured.^25^

### Determination of Elastase

A 1000 μL portion was taken from the mother flasks and then centrifuged for 1.5 min at 13,000 rpm, and the supernatant was separated. Subsequently, 100 μL of the supernatant from each of the cultures was placed, and 5 mg of elastin-Congo red from Sigma-Aldrich and 900 μL of pH 7.5 buffer were added. They were incubated for 2 h at 37 °C and centrifuged at 13,500 rpm for 5 min, and absorbance was measured at 495 nm.^26^

### Molecular Docking

The 2D structure of curcumin and 5-bromoindole-3-carboxaldehyde was drawn with ChemSketch ACD/Laboratories, and protonation states were calculated at pH 7.4 with MarvinSketch Chemaxon. The 3D structure was obtained and optimized to a semiempirical PM3 level with Spartan 08 V1.2.0. The crystals of the LasR, PQS, and RhlR proteins were obtained from the Protein Data Bank repository with the codes 3IX3, 4JVI, and 8DQ0, respectively. The preparation of the proteins was carried out in Molegro Virtual Docker, the water molecules were removed, the druggable cavities were detected, and the ligand-binding site was selected for molecular docking studies with a search sphere of a 10 Å radius and a grid of 0.30 Å, with the coordinates *X*: 10.54, *Y*: 4.32, and *Z*: 20.98 for LasR, *X*: −34.71, *Y*: 56.02, and *Z*: 9.17 for PQS, and *X*: 116.01, *Y*: 96.69, and *Z*: 137.48 for RhlR.^27–29^

The validation of the molecular docking method (redocking) was carried out using 12 methods based on the combinations of 4 scoring functions (MolDock Score, MolDock Score GRID, Plant Score, and Plant Score GRID) and 3 search algorithms (MolDock Optimizer, MolDock SE, and Iterated Simplex). In each method, they consist of a scoring function and a search algorithm (MolDock Score-MolDock Optimizer, MolDock Score-MolDock SE, MolDock Score-Iterated Simplex, MolDock Score GRID-MolDock Optimizer, MolDock Score GRID-MolDock SE, MolDock Score GRID-Iterated Simplex, Plant Score-MolDock Optimizer, Plant Score-MolDock SE, Plant Score-Iterated Simplex, Plant Score GRID-MolDock Optimizer, Plant Score GRID-MolDock SE, and Plant Score GRID-Iterated Simplex); 10 runs were performed, a maximum of 1500 interactions, and an initial population of 50 poses.^27,28^ Of the 12 redocking methods, the method for performing molecular docking studies of each protein, which consists of the scoring function and search algorithm, was chosen considering an rmsd value ≤ 2.0 as an inclusion criterion,^30^ obtained for a coupled ligand with respect to the position of the cocrystallized ligand. The binding energy was obtained in the online server PROtein binDIng enerGy prediction (PRODIGY),^31^ and the interactions were visualized with the BIOVIA Discovery Studio program.^32^

### Molecular Dynamics Simulations

To evaluate the dynamic stability and conformational flexibility of the protein–ligand complexes from molecular docking under physiological conditions, molecular dynamics (MD) simulations were performed using GROMACS 2024-3 software.^33,34^ The force field used within MD considered all atoms, and it was consistent with the CHARMM36 parameter set.^35^ For proteins LasR, PQS, and RHIR, the parametrization for this force field is straightforward, and the topology is automatically generated by GROMACS. In the case of curcumin and 5-bromoindole-3-carboxaldehyde, the generation of topologies and the assignment of parameters were conducted by the CGenFF Web App by SilcsBio.^36^

After topologies and force field parameters were generated and validated, each of the protein–ligand systems was placed in the center of a simulation box and subsequently solvated with liquid water using the TIP3P potential.^37^ Also, Cl^–^ and Na^+^ ions were added in a proportion that would neutralize the overall charge of the system and obtain a final salt concentration of 100 mM. Both the energy minimization to avoid spurious contacts and the equilibration of systems were carried out according to the protocol and parameters set outlined in ref 38.

For production and obtaining observables, MD simulations were performed in the isothermal–isobaric ensemble (NPT). The Nose–Hoover thermostat and Parrinello–Rahman barostat were used to keep a constant temperature of 310 K and a pressure of 1 bar, respectively.^39^ The time interval of Δ*t* = 0.002 ps was established to integrate the equations of motion. Then, 200 ns trajectories were generated for each system. Hydrogen bonding interactions, root-mean-square deviation (rmsd), root-mean-square fluctuation (RMSF), and binding energy were calculated from MD simulations.

To obtain an appropriate estimate of the binding energy for the protein–ligand complex, we used the Molecular Mechanics Poisson–Boltzmann Surface Area (MM/PBSA) method.^40^ In broad terms, MM/PBSA takes into account the gas-phase free energy contribution (Δ*E*_MM_), the solvation free energy (Δ*E*_solv_), and the conformational entropy (−*T*Δ*S*):

$$\Delta G_{bind}=\Delta E_{MM}+\Delta E_{solv}-T\Delta S$$

In this framework, the gas-phase free energy considers both electrostatic and van der Waals contributions (Δ*E*_MM_ = Δ*E*_ele_ + Δ*E*_vdw_), while the solvation free energy comprises both polar and nonpolar contributions (Δ*E*_solv_ = Δ*E*_pol_ + Δ*E*_non‑pol_). However, the entropy term (−*T*Δ*S*) from MD simulations remains a challenging task. Within the MMPBSA framework, the methods for estimating binding entropy are based on both the interaction entropy (IE) approach and the second-order cumulant (C2) approximation, which relies on the interaction energy standard deviation (σ_IE_) through the expression −*T*Δ*S* = σ_IE_^2^/*RT*. In a comprehensive study on the convergence of these binding entropies, Ekberg and Ryde^41^ showed that the IE approach suffers serious convergence problems due to the use of exponential averaging, which is intrinsically poorly conditioned. When σ_IE_ > 3.6 kcal/mol, the estimates become practically impossible to converge and therefore unreliable. Entropy estimation is further complicated by the choice of sampling size and frequency: although frequent sampling has been proposed to improve convergence, it is highly inefficient and introduces strong correlations between energies, and increasing the number of samples often leads to a systematic rise in the estimated entropy, indicating poor convergence and limiting the reliability of these values. Therefore, the omission of entropy terms becomes necessary for systems with σ_IE_ > 3.6 kcal/mol, as observed in our case (see Table 9). Including such poorly converged entropy contributions would introduce significant uncertainty into the calculated binding free energy. Although neglecting entropy affects the absolute accuracy of the calculated binding free energy, in these cases the IE estimates are numerically unrealistic, making the inclusion more detrimental than their exclusion.

Thus, the binding energies obtained from the MMPBSA approximation should be interpreted as reliable estimates, suitable for comparative analysis rather than quantitatively accurate absolute free energies. MMPBSA calculations also exploit the trajectories generated from the MD simulations.

### Statistical Analysis

Two-way analysis of variance (ANOVA) with replication was performed to evaluate the effect of different treatments on bacterial strains Pa01, Pa14, and ATCC27853. The treatments included 5-bromoindole-3-carboxaldehyde at three different concentrations (30 μg/mL, 50 μg/mL, and 100 μg/mL) and curcumin at two different concentrations (1 μg/mL and 5 μg/mL). Both the ANOVA analysis and the calculation of standard deviation (SD) were performed using IBM SPSS Statistics for Windows, version 21.0 (IBM Corp., Armonk, NY). Post hoc comparisons were performed using Tukey’s honestly significant difference (HSD) test with *p* < 0.05.

## Results

### Experimental Evaluation

In the present study, the effectiveness of two compounds, 5-bromoindole-3-carboxaldehyde and curcumin, at different concentrations in the inhibition of pyocyanin, alginate, elastase, and protease quorum-dependent virulence factors and the minimal inhibitory concentration (MIC) in three *P. aeruginosa* strains was evaluated: Pa01, Pa14, and ATCC 27853.

### Minimal Inhibitory Concentration (MIC)

The MIC values evaluated for curcumin showed no inhibitory activity in the 15.62–250 μg/mL range. Growth inhibition was evident only at concentrations ≥500 μg/mL and up to 1000 μg/mL. In contrast, 5-bromoindole-3-carboxaldehyde showed significant inhibitory activity in all strains starting at a concentration of 125 μg/mL, without a progressive increase in concentration-dependent inhibition (Table 1).

**Table 1:** Minimum Inhibitory Concentrations (MIC) of Curcumin and 5-Bromoindole-3-carboxaldehyde against Three *Pseudomonas aeruginosa* Strains

compound	concentration (μg/mL)	Pa01	Pa14	ATCC 27853
curcumin	15.62			
	31.25			
	62.5			
	125			
	250			
	500	MIC	MIC	MIC
	1000	MIC	MIC	MIC
5-bromoindole-3-carboxaldehyde	62.5			
	125	MIC	MIC	MIC
	250	MIC	MIC	MIC
	500	MIC	MIC	MIC
	1000	MIC	MIC	MIC

This indicates no inhibition at the tested concentration. The MIC corresponds to the lowest concentration showing complete inhibition.

Pyocyanin:bromoindole: strain Pa01 showed significant inhibition at concentrations of 30 μg/mL (85.42%), 50 μg/mL (87.77%), and 100 μg/mL (92.5%). The Pa14 strain also presented a reduction, although smaller, with values of 60.3%, 61.67%, and 67.65% at the same concentrations, respectively. Strain ATCC 27853 showed lower inhibition at 30 μg/mL (8.7%) and 50 μg/mL (13.05%) but higher inhibition at 100 μg/mL (72.47%). Regarding the Pa14 strain at the same concentration, although the Pa01 strain was the one that produced the highest concentration of pyocyanin in its control, it was the strain in which the molecule had the highest activity at all the concentrations studied. On the other hand, the control production of the Pa14 strains and the ATCC 27853 strain varies by 0.001, showing greater activity in the Pa14 strain at concentrations of 30 and 50 μg/mL but not for the concentration of 100 μg/mL, where greater inhibition is seen in strain ATCC 27853.

When curcumin was added, strain Pa01 showed an inhibition of 60.68% at 1 μg/mL and 90.63% at 5 μg/mL. The Pa14 strain presented inhibitions of 19.12% at 1 μg/mL and 55.89% at 5 μg/mL. The ATCC 27853 strain showed inhibitions of 81.16% at 1 μg/mL and 100% at 5 μg/mL. Although total inhibition is achieved in strain ATCC 27853, it is the one that showed the lowest pyocyanin production; however, it is the strain with the highest activity, followed by Pa01 and Pa14, respectively (Chart 1).

**Chart 1:**
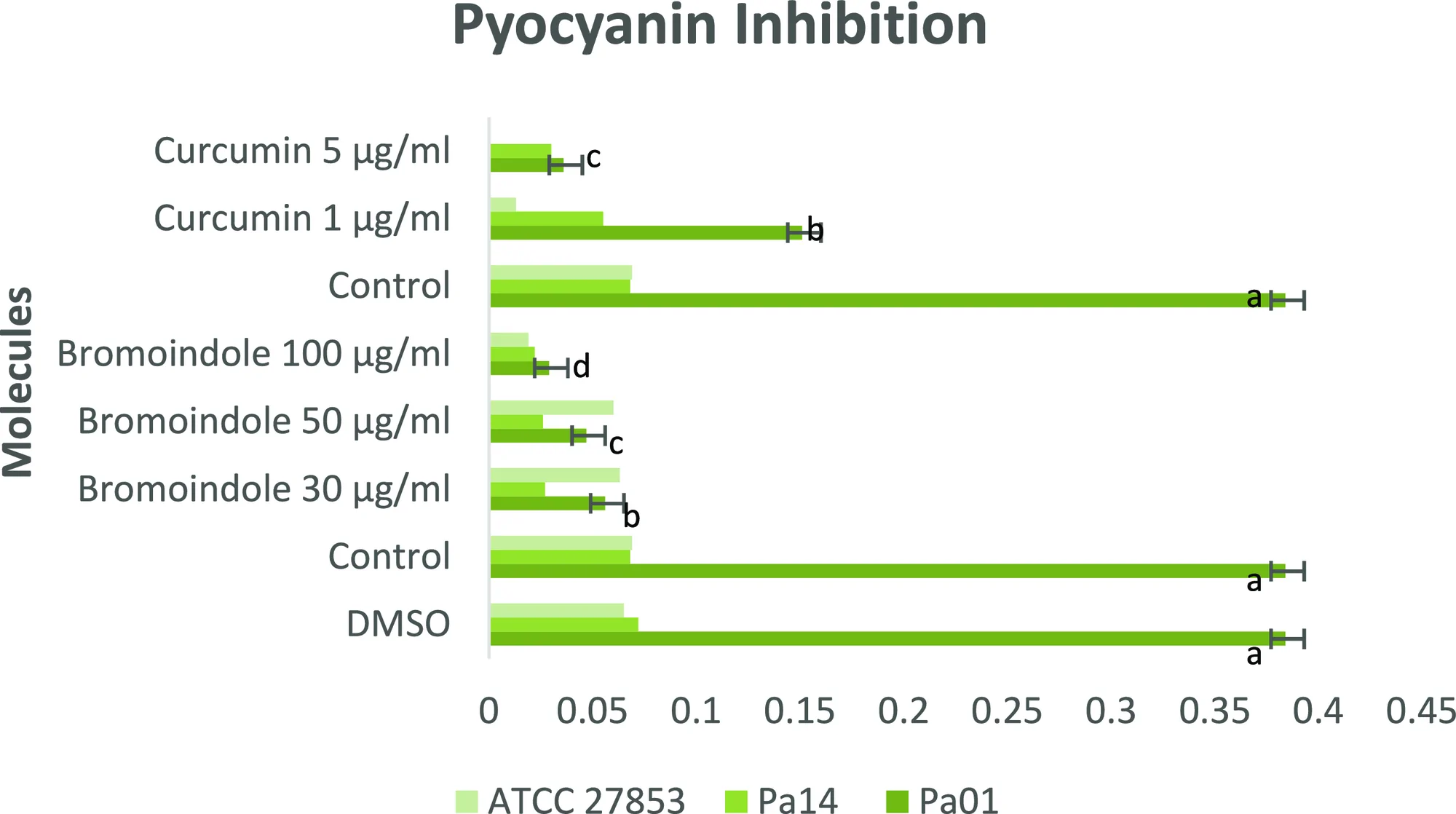
Pyocyanin Inhibition by Curcumin and 5-Bromoindole-3-carboxaldehyde at Different Concentrations; Data Are Presented as Mean ± SD (*n* = 3); Statistical Differences among Groups Were Determined by ANOVA Followed by Tukey’s HSD Post Hoc Test (*p* < 0.05)

ANOVA revealed significant differences in pyocyanin production among treatment groups in all strains (*p* < 0.001). Tukey HSD analysis showed that 5-bromoindole-3-carboxaldehyde significantly reduced pyocyanin production relative to control, with concentration-dependent inhibition observed in Pa01 and Pa14. Curcumin significantly reduced pyocyanin production in all evaluated strains, as demonstrated by ANOVA (*p* < 0.001). Tukey HSD analysis confirmed significant differences between treated and control groups, with greater inhibition observed at 5 μg/mL.

Elastase:bromoindole: for strain Pa01, a significant inhibition was observed with values of 69.77% at 30 μg/mL, 70.64% at 50 μg/mL, and 79.85% at 100 μg/mL. The Pa14 strain showed a variable behavior since, when adding 30 μg/mL, no inhibition was observed; on the contrary, it seemed that it favored the expression of the virulence factor with 21.11% more compared to the control. An inhibition of 30.42% was shown at 50 μg/mL and 32.99% at 100 μg/mL. The ATCC 27853 strain showed an initial inhibition at 100 μg/mL (31.35%) since, as happened with the Pa14 strain, at concentrations of 30 and 50, greater production of the virulence factor was shown, which suggests that at low concentrations, it does not inhibit, but it does stimulate production. Therefore, the molecule showed a high inhibitory effect at all concentrations studied in the Pa01 strain; for the Pa14 strain, it is the second in terms of activity, and in the ATCC 27853 strain, it showed the least activity.

The Pa01 strain presented inhibitions when adding curcumin of 3.6% at 1 μg/mL and 32.08% at 5 μg/mL. Strain Pa14 showed minor inhibitions of 5.93% at 1 μg/mL and 56.19% at 5 μg/mL. The ATCC 27853 strain presented inhibitions of 5.65% at 1 μg/mL and 18.5% at 5 μg/mL. The molecule at 1 μg/mL presented the least inhibition in the Pa01 strain and greater activity at both concentrations in the Pa14 strain. Chart 2.

**Chart 2:**
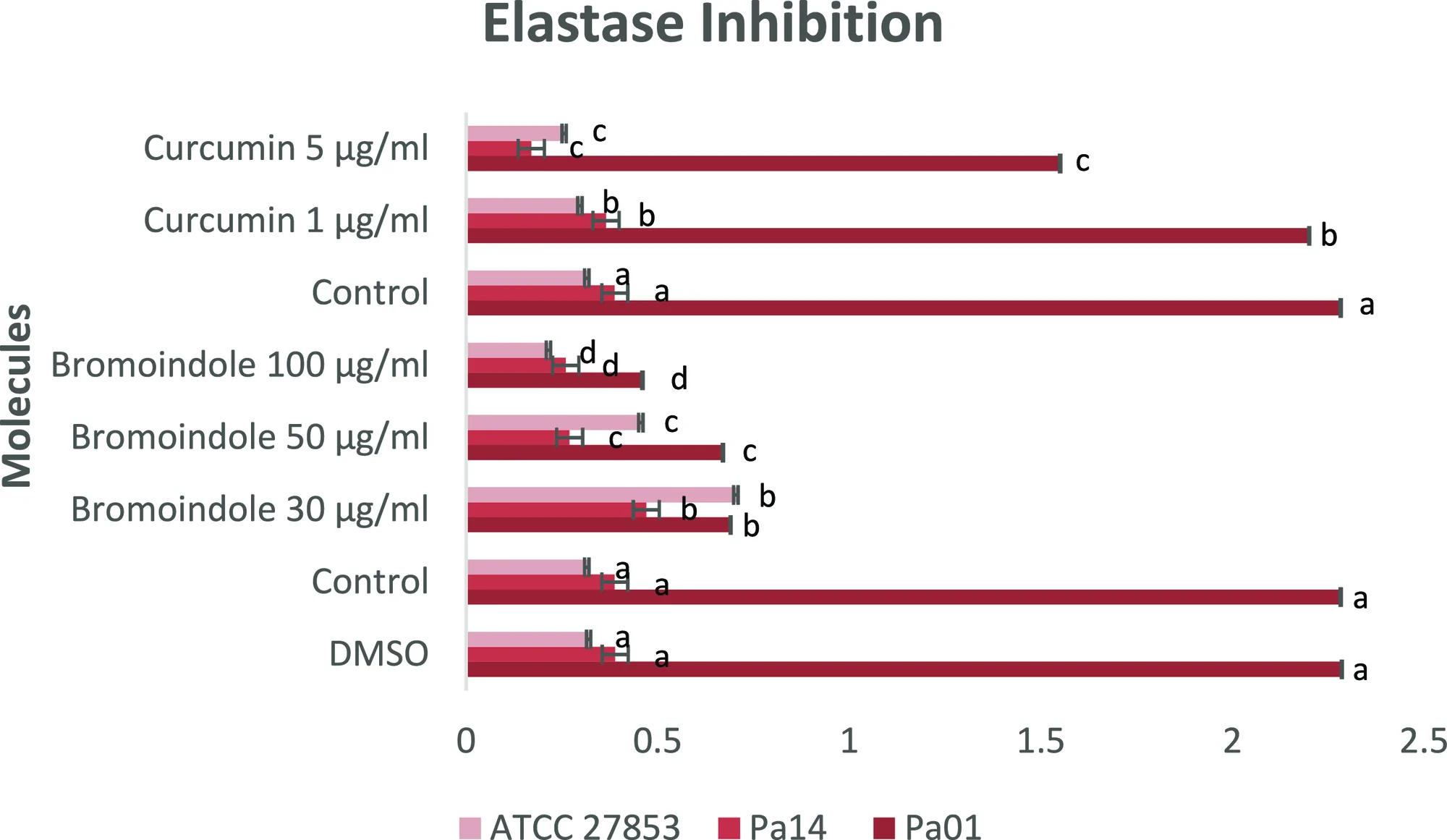
Elastase Inhibition by Curcumin and 5-Bromoindole-3-carboxaldehyde at Different Concentrations; Data Are Presented as Mean ± SD (*n* = 3); Statistical Differences among Groups Were Determined by ANOVA Followed by Tukey’s HSD Post Hoc Test (*p* < 0.05)

Significant differences in elastase activity were detected across treatment groups (*p* < 0.001). Tukey’s post hoc analysis confirmed significant inhibition at higher concentrations of 5-bromoindole-3-carboxaldehyde, particularly at 100 μg/mL, although strain-dependent responses were observed at lower concentrations. ANOVA showed significant differences in elastase activity following curcumin treatment (*p* < 0.001). Tukey’s post hoc analysis indicated that 5 μg/mL produced stronger inhibition than 1 μg/mL in most strains.

Protease:bromoindole: inhibition in strain Pa01 was 92.51% at 30 μg/mL, 92.8% at 50 μg/mL, and 89.92% at 100 μg/mL. It is the strain where, at low concentrations of 30 μg/mL and 50 μg/mL, a considerable inhibition is seen; however, at 100 μg/mL, no greater inhibition is seen; on the contrary, it seems that it decreases by about 3% compared to concentration no. 2. Strain Pa14 showed variable inhibition, with an initial increase at 30 μg/mL (66.6%), a reduction at 50 μg/mL (24.25%), and almost complete inhibition at 100 μg/mL (96.22%). The ATCC 27853 strain presented an inhibition of 85.56% at 50 μg/mL and 97.48% at 100 μg/mL, although at 30 μg/mL, it also showed an increase in the expression of the virulence factor. However, for this virulence factor at concentrations of 30 and 50 μg/mL, it is more effective in the Pa01 strain, while at the concentration of 100 μg/mL, the strain that showed the greatest activity was ATCC 27853, and the one that showed the least activity at the same concentration was Pa01.

For curcumin, the inhibition in strain Pa01 was 79.83% at 1 μg/mL and 87.04% at 5 μg/mL. The Pa14 strain presented inhibitions of 1.52% at 1 μg/mL and 38.64% at 5 μg/mL. The ATCC 27853 strain showed inhibitions of 41.51% at 1 μg/mL and 71.12% at 5 μg/mL. The strain that showed the greatest inhibition of this virulence factor is Pa01, followed by ATCC 27853 and the one that showed the least activity against the molecule was Pa14 (Chart 3).

**Chart 3:**
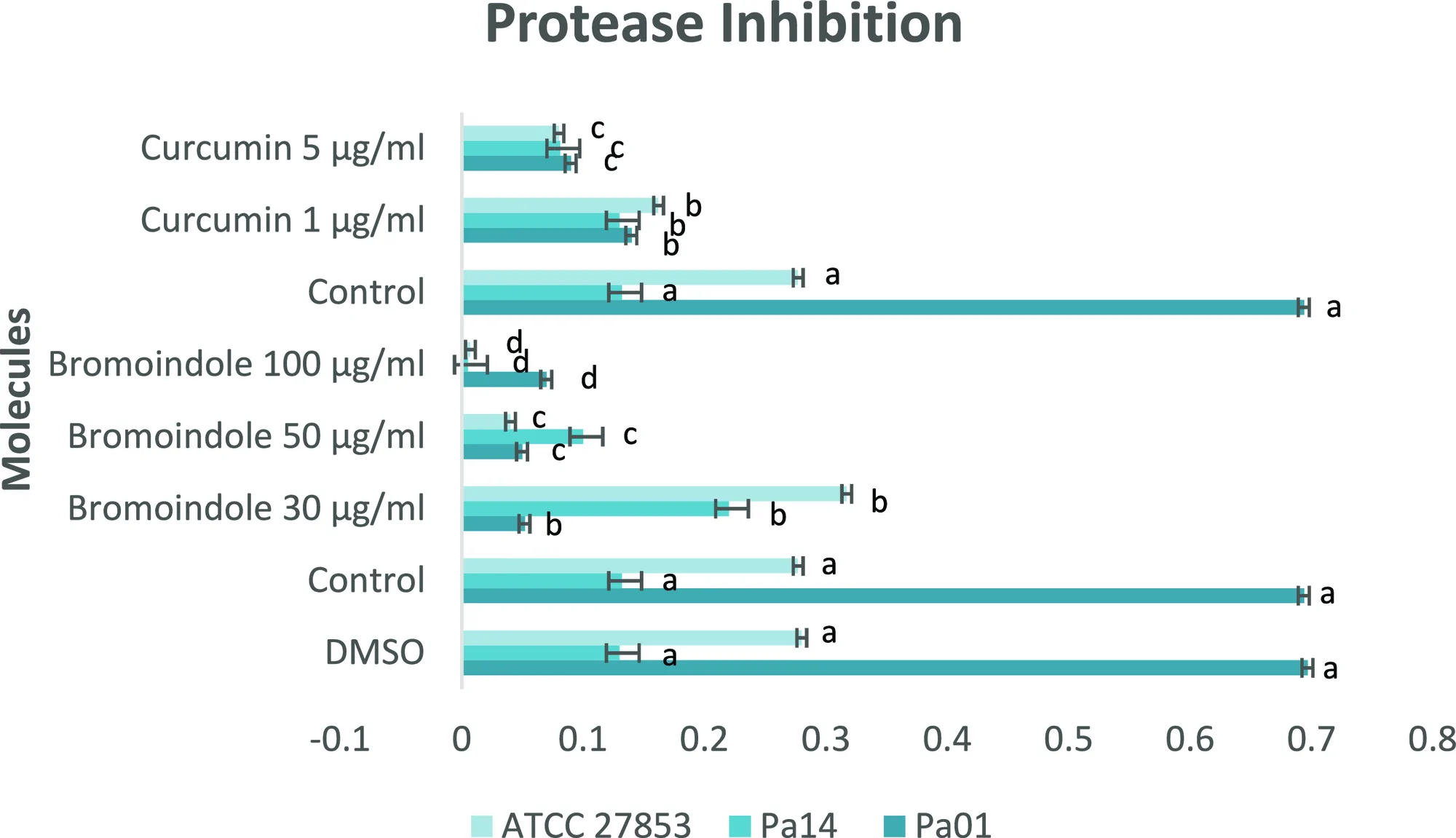
Protease Inhibition by Curcumin and 5-Bromoindole-3-carboxaldehyde at Different Concentrations; Data Are Presented as Mean ± SD (*n* = 3); Statistical Differences among Groups Were Determined by ANOVA Followed by Tukey’s HSD Post Hoc Test (*p* < 0.05)

Protease production was significantly altered by curcumin treatment (*p* < 0.001). Tukey HSD analysis revealed significant inhibition relative to control, particularly at 5 μg/mL, indicating dose-dependent antivirulence activity. Protease activity differed significantly among treatment groups according to ANOVA (*p* < 0.001). Tukey HSD demonstrated that 5-bromoindole-3-carboxaldehyde significantly inhibited protease production compared with controls, with the strongest effects generally observed at 50–100 μg/mL.

Alginate:bromoindole: strain Pa01 showed an inhibition of 35.87% at 30 μg/mL, 48.4% at 50 μg/mL, and 61.4% at 100 μg/mL. The Pa14 strain presented inhibitions of 16.27% at 30 μg/mL, 18.17% at 50 μg/mL, and 40.67% at 100 μg/mL. Strain ATCC 27853 showed an initial increase in alginate production at 30 μg/mL (12.22%) but a significant inhibition at 50 μg/mL (52.78%) and 100 μg/mL (76.16%). The molecule was more active in strains Pa01 and ATCC 27853 at concentrations of 50 and 100 μg/mL, where the greatest activity was seen in the latter strain. For Pa14, it showed the least activity, but with significant inhibition.

With curcumin, strain Pa01 showed inhibitions of 37.91% at 1 μg/mL and 48.26% at 5 μg/mL. The Pa14 strain presented inhibitions of 32.3% at 1 μg/mL and 41.63% at 5 μg/mL. The ATCC 27853 strain showed inhibitions of 2.78% at 1 μg/mL and 15% at 5 μg/mL (Chart 4).

**Chart 4:**
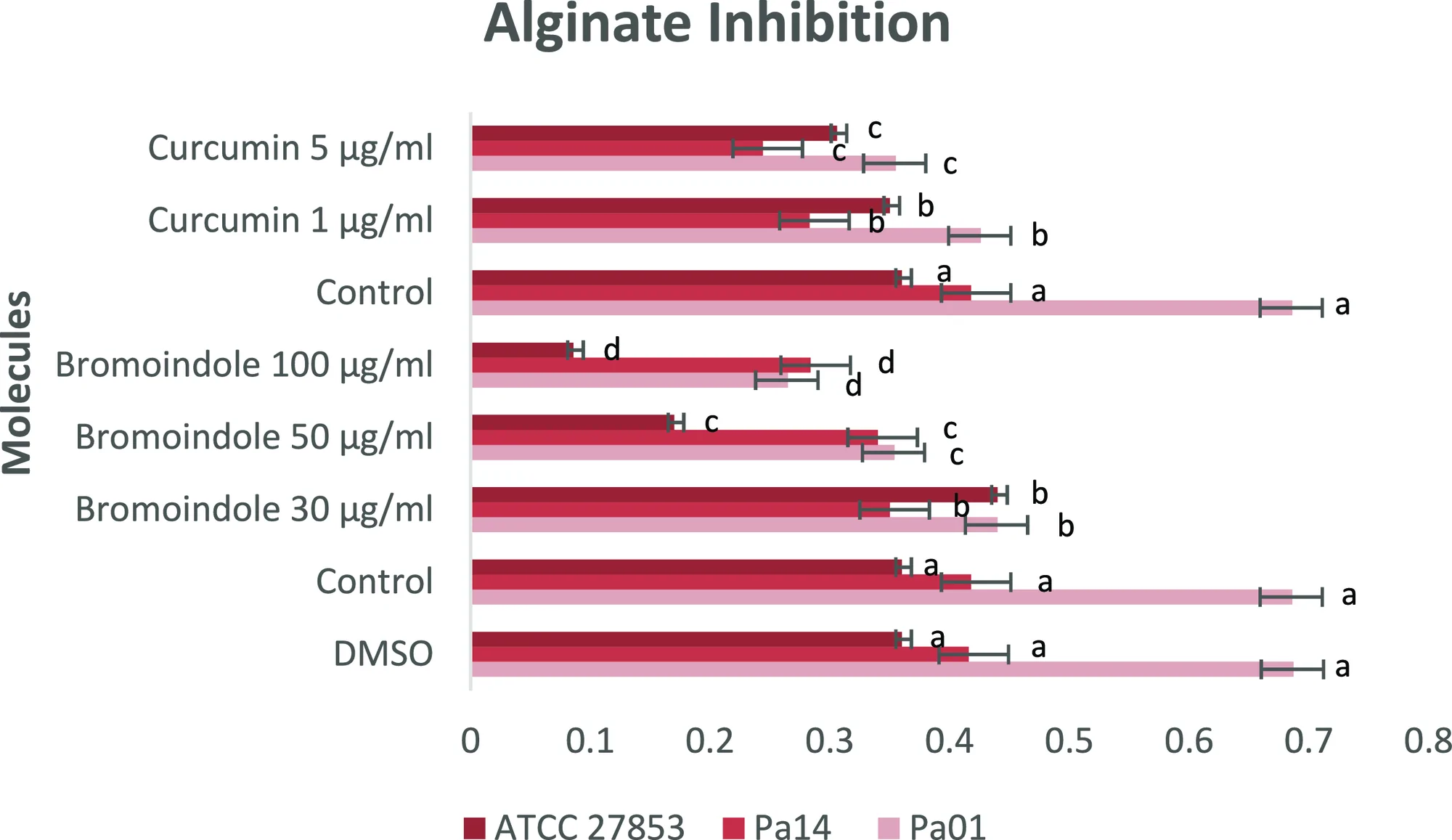
Alginate Inhibition by Curcumin and 5-Bromoindole-3-carboxaldehyde at Different Concentrations; Data Are Presented as Mean ± SD (*n* = 3); Statistical Differences among Groups Were Determined by ANOVA Followed by Tukey’s HSD Post Hoc Test (*p* < 0.05)

Alginate production was significantly affected by treatment in all strains (*p* < 0.001). Tukey’s HSD analysis confirmed significant reductions in alginate production at increasing concentrations of 5-bromoindole-3-carboxaldehyde, consistent with concentration-dependent inhibition. Curcumin significantly modulated alginate production across all strains according to ANOVA (*p* < 0.001). Tukey’s HSD confirmed significant reductions in alginate levels in treated groups, with 5 μg/mL generally showing the greatest inhibitory effect.

Table 2 shows the results in percentage and inhibition concentration of the virulence factors: pyocyanin, elastase, protease, and alginate studied with bromoindole. Table 3 shows the results in percentage and inhibition concentration of the virulence factors with curcumin.

**Table 2:** Inhibition Percentages of 5-Bromoindole-3-carboxaldehyde on Quorum-Dependent Virulence Factors

virulence factors																
strain/ [ ]	pyocyanin				elastase				protease				alginate			
	control	30 μg/mL	50 μg/mL	100 μg/mL	control	30 μg/mL	50 μg/mL	100 μg/mL	control	30 μg/mL	50 μg/mL	100 μg/mL	control	30 μg/mL	50 μg/mL	100 μg/mL
Pa01	0 (0.384)	85.42 (0.56)	87.77 (0.047)	92.5 (0.029)	0 (2.282)	69.77 (0.69)	70.64 (0.67)	79.85 (0.46)	0 (0.694)	92.51 (0.052)	92.8 (0.05)	89.92 (0.07)	0 (0.686)	35.87 (0.44)	48.4 (0.354)	61.4 (0.265)
Pa14	0 (0.068)	60.3 (0.027)	61.67 (0.026)	67.65 (0.022)	0 (0.388)	+21.11 (0.47)	30.42 (0.27)	32.99 (0.26)	0 (0.132)	+66.6 (0.22)	24.25 (0.1)	96.22 (0.005)	0 (0.418)	16.27 (0.35)	18.17 (0.34)	40.67 (0.284
ATCC 27853	0 (0.069)	8.7 (0.063)	13.05 (0.06)	72.47 (0.019)	0 (0.319)	+121.1 (0.708)	+44.22 (0.46)	31.35 (0.219)	0 (0.277)	+14.44 (0.317)	85.56 (0.04)	97.48 (0.007)	0 (0.36)	+12.22 (0.44)	52.78 (0.17)	76.16 (0.086)

**Table 3:** Inhibition Percentages of Curcumin on Quorum-Dependent Virulence Factors

virulence factors												
strain/[ ]	pyocyanin			elastase			protease			alginate		
	control	1 μg/mL	5 μg/mL	control	1 μg/mL	5 μg/mL	control	1 μg/mL	5 μg/mL	control	1 μg/mL	5 μg/mL
**Pa01**	0 (0.384)	60.68 (0.151)	90.63 (0.036)	0 (2.282)	3.6 (2.2)	32.08 (1.55)	0 (0.695)	79.83 (0.14)	87.04 (0.09)	0 (0.686)	37.91 (0.426)	48.26 (0.355)
**Pa14**	0 (0.068)	19.12 (0.055)	55.89 (0.03)	0 (0.388)	5.93 (0.365)	56.19 (0.17)	0 (0.132)	1.52 (0.13)	38.64 (0.081)	0 (0.418)	32.3 (0.283)	41.63 (0.244)
**ATCC 27853**	0 (0.069)	81.16 (0.013)	100 (0)	0 (0.319)	5.65 (0.301)	18.5 (0.26)	0 (0.277)	41.51 (0.162)	71.12 (0.08)	0 (0.36)	2.78 (0.35)	15 (0.306)

## Computational Studies

Molecular and cheminformatics properties were obtained using Molinspiration, Molsoft, ChemSketch, swissADME, and Osiris Property Explorer simulators. The property values of curcumin and 5-bromoindole-3-carboxaldehyde (Figure 1) are summarized in Table 3. Lipinski’s rules indicate that a compound must meet the following criteria to have good bioavailability and to be preferably administered orally: (a) log *P* value < 5; (b) TPSA < 140 Å; (c) molecular weight <500 Da; (d) hydrogen bond donors (−NH, –OH) < 5; and (e) hydrogen bond acceptors (N, O) < 10.^42^ The Lipinski’s rule of 5 aids in differentiating between drug-like and nondrug-like features and forecasts a high likelihood of success or failure due to a molecule’s drug-likeness.^43^ As can be seen in the table, curcumin and 5-bromoindole-3-carboxaldehyde do not present violations of Lipinski’s rules; this would indicate that both compounds are candidates for oral administration, which is an advantage in antibacterial therapy.

**Figure 1:**
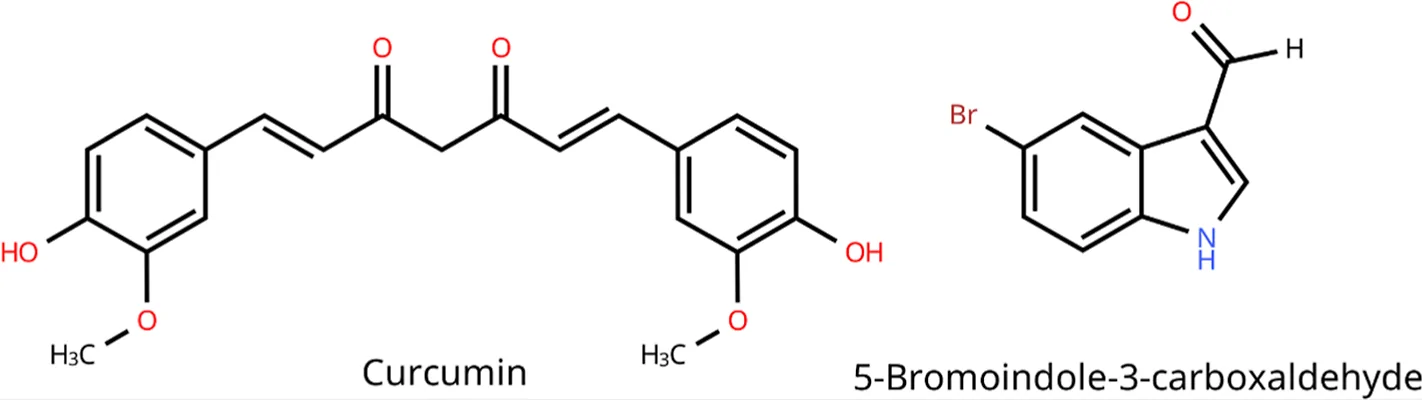
Structure of curcumin and carboxaldehyde.

The characteristics to highlight include solubility; the log *P* is relevant to drug discovery, as it is used to describe lipophilicity, which influences drug targets. A relatively high lipophilicity results in reduced aqueous solubility and increased likelihood of metabolic instability;^44^ as seen in Table 4, curcumin has a log *P* of 2.30 and 5-BIC of 2.66; therefore, both compounds are water-soluble. The PSA indicates the polar surface area of a molecule and the ease with which it can penetrate the cell, for which it must be less than 140 Å; curcumin has a PSA of 93.07 and 5-BIC of 32.86, and this is indicative that the molecules could easily penetrate the cell membrane to carry out an effect on the cellular organelles. The presence of a hydrogen bond donor generally implies that a hydrogen bond acceptor is also present; these properties indicate electrostatic interactions resulting from aligned hydrogen bond donors and acceptors, which is useful in the recognition of molecular targets.^45^ The topological descriptors of hydrophobicity (log *P*), molar refractivity (MR), polarizability (*P*), and polar surface area (PSA) play an important role when estimating their activity and are generally related to the drug-active site interaction. Compounds with a water-soluble log *P* usually present greater biological activity. Likewise, molar refractivity values close to 70 coincide with an increase in activity. Low polarizability (less than 50) indicates that the compound is specific; that is, it does not have the capacity to bind to several ligands, but rather its specificity for a receptor or target site increases. The data in Table 4 indicate that the presence of polar atoms in the molecule (but not polarizable) is necessary for these to be active. These results strengthen the proposal to be administered orally.^46^

**Table 4:** Molecular and Chemoinformatic Properties of Curcumin and 5-Bromoindole-3-carboxaldehyde^a^

properties	curcumin	5-bromoindole-3-carboxaldehyde
MF	C_21_H_20_O_6_	C_9_H_6_BrNO
MW	368.3799	224.05404
no. HBA	6	2
no. HBD	2	1
PSA	93.07	32.86
log *P*	2.30	2.66
δ	1.279 ± 0.06 g/cm^3^	1.727 ± 0.06 g/cm^3^
ST	54.3 ± 3.0 dyn/cm	63.5 ± 3.0 dyn/cm
polarizability	41.24 ± 0.5 × 10^–24^ cm^3^	20.99 ± 0.5 × 10^–24^ cm^3^
MR	104.04 ± 0.3 cm^3^	52.97 ± 0.3 cm^3^
MV	287.8 ± 3.0 cm^3^	129.7 ± 3.0 cm^3^
Nrotb	8	1
drug-likeness	–3.95	–6.29
Lipinski’s rule violations	0	0
SMILES	COc2cc(C=CC(–O)CC(–O)C=Cc1ccc(O) c(OC)c1)ccc2O (E/Z)-curcumin	[H]C(–O)c1c[nH]c2ccc(Br)cc12

a MF = molecular formula, MW = molecular weight (Da), HBA = hydrogen bond acceptors, HBD: hydrogen bond donors, PSA = polar surface area (Å^2^), log *P* = partition coefficient, δ = density (g/cm^3^), ST = surface tension, MR = molar refractivity (cm^3^), nrotb = no. of rotatable bonds, MV = molar volume (cm^3^), and SMILES = simplified molecular input line entry system.

Molinspiration and Osiris Property Explorer simulators were used to predict the bioactivity of the compounds against important drug target sites and to predict their potential tumorigenic, irritant, reproductive, and mutagenic risks; these results are found in Table 5. The bioactivity and biophysical properties indicate that curcumin is the compound with the greatest pharmacological interest, mainly at the target sites of nuclear receptor ligands, and as an enzyme inhibitor, it is important that the values have a positive value to consider them bioactive at a target site.

**Table 5:** Prediction of Bioactivity and Toxicity of Compounds **1** and **2** ^a^

compound	NCL	IE	GPCR	KI	PI	RE	T	M	I
curcumin	0.12	0.08	–0.06	–0.26	–0.14	no	no	no	no
5-bromoindole-3-carboxaldehyde	–1.22	–0.53	–0.77	–0.57	–1.53	no	no	no	no

a NCL = nuclear receptor ligand, IE = enzyme inhibitor, GPCR = GPCR ligand, KI = kinase inhibitor, RE = reproductive effective, T = tumorigenic, M = mutagenic, and I = irritant.

The greatest failure in the drug discovery process is toxicity, and according to the prediction, curcumin and 5-bromoindole-3-carboxaldehyde should not present tumorigenic, irritant, reproductive, or mutagenic effects. The inhibition predictions of curcumin and 5-bromoindole-3-carboxaldehyde with CYP450 enzymes are made based on the structural formula of the compounds; this gives us an approximation of the metabolism of each molecule. According to Table 5, curcumin inhibits the CYP3A4 and CYP1A2 isoforms, while carboxaldehyde only seems to inhibit CYP1A2; it is important to mention that the greater or lesser effect of an enzyme inhibitor will depend on factors such as the dose of the inhibitor and its capacity to bind to the enzyme. In the predictions made, it is not indicated whether the compounds are mild or potent inhibitors, but this data should be taken into account to consider possible drug–drug interactions (DDIs), as well as the doses that could be administered to a patient.^47^

On the basis of the combined results of the cheminformatics properties, curcumin is the most promising compound to have the desired biological activity, which would be reflected in a possibly better availability and distribution of the compound in the body; this is corroborated by the absorption data in Table 6.

**Table 6:** Data of ADME Processes^a^

ADMET	curcumin	5-bromoindole-3-carboxaldehyde	
absorption	WS	–4.048	–2.891
	IA (%)	87.577	92.361
	SP	–2.755	–2.425
	SGP	yes	yes
	P-GP-1-In	yes	no
distribution	BBBP	–0.511	yes
	CNSP	–3.07	–1.758
metabolism	CYP3A4 inhibitor CYP1A2 inhibitor	yes	no
		yes	yes
excretion	TC	0.06	0.204

a WS: water solubility (log mol/L), IA: intestinal absorption, SP: skin permeability (log Kp), SGP: *P*-glycoprotein substrate, *P*-GP-1-In: *P*-glycoprotein I inhibitor, BBBP: blood–brain barrier permeability (log BB), CNSP: CNS permeability (log PS), and TC: total clearance (log mL/min/kg).

Metabolic Stability of Curcumin and 5-Bromoindole-3-carboxaldehyde^48^

The metabolism of curcumin in complex biological environments exhibits marked instability, which is a significant limitation for its pharmacological development. To mitigate this, curcumin requires formulation strategies (nanoparticles or liposomes) to reduce its rate of degradation.^48,49^ Under physiological conditions (pH close to 7.4), the molecule undergoes rapid nonenzymatic degradation, primarily through oxygen-dependent autoxidation.

The presence of serum proteins, such as albumin (in its hydrophobic cavities and Sudlow binding site II), acts as a protective shield that partially modulates the metabolic instability of curcumin, increasing its half-life from a few minutes to approximately 1–2 h. This occurs because the molecule becomes “trapped” within the protein, which removes it from the alkaline aqueous environment that promotes its degradation, in addition to limiting its freedom of movement, hindering the structural changes necessary for its autoxidation. Albumin acts as a slow-release system, maintaining a constant concentration of free curcumin that can interact with microorganisms while protecting the rest of the payload against immediate chemical degradation by inhibiting the formation of the endoperoxide intermediate, which is the critical step for the fragmentation of the molecule. Due to these protein interactions that limit its degradation, the antimicrobial effects of curcumin can be attributed to both the original molecule and its metabolites.^48^

In *in vivo* systems, curcumin undergoes extensive metabolic biotransformation, primarily through glucuronidation and sulfation reactions, generating more stable metabolites (curcumin glucuronide and curcumin sulfate) that generally have lower biological activity. Furthermore, these metabolites are much more polar (hydrophilic), which facilitates their rapid renal and biliary excretion, drastically reducing the compound’s residence time in the body.^50^ This indicates that curcumin has low overall metabolic stability, dependent on the biological microenvironment.

In contrast, chemoinformatic analysis of the chemical structure of 5-bromoindole-3-carboxaldehyde reveals relevant characteristics. For example, this compound belongs to the family of indole derivatives, which have demonstrated greater structural stability in biological systems and relative resistance to rapid degradation processes compared to polyphenols such as curcumin.^51^ The bromine atom acts as an electron shield, hindering the hydroxylation of the ring by cytochrome P450 enzymes, thus prolonging the compound’s half-life in the body and allowing for more effective dosing.

The ADME analysis performed in this study suggests that 5-bromoindole-3-carboxaldehyde exhibits high intestinal absorption (≈92%) and distribution capacity, including permeability across the blood–brain barrier, which indirectly supports its persistence in complex biological environments. Furthermore, the predicted limited interaction with cytochrome P450 enzymes (primarily CYP1A2) indicates a potentially more stable metabolic profile or one less susceptible to rapid biotransformation compared to curcumin.

The presence of the bromine substituent on the indole ring may further contribute to its stability by modifying electronic and steric properties that hinder oxidative processes and increase lipophilicity, thus promoting its persistence in complex biological environments.

## Molecular Docking Studies

We performed molecular docking studies to understand the binding interactions of curcumin and 5-bromoindole-3-carboxaldehyde with the LasR, PQS, and RhlR proteins of the quorum sensing of *Pseudomonas aeruginosa*. The molecular docking protocols were validated with redocking, coupling the cocrystallized ligands in the proteins obtained in the Protein Data Bank using 12 methods from the combination of search algorithms and scoring functions, which are described in the materials and methods section. Figure 2 shows the results of the redocking of the LasR, PQS, and RhlR proteins with the cocrystallized ligands, as well as the method that was selected to perform the molecular docking of the two compounds under study from an rmsd less than 2 Å, which indicates that these methods can predict the experimental binding mode in the quorum-sensing proteins.

**Figure 2:**
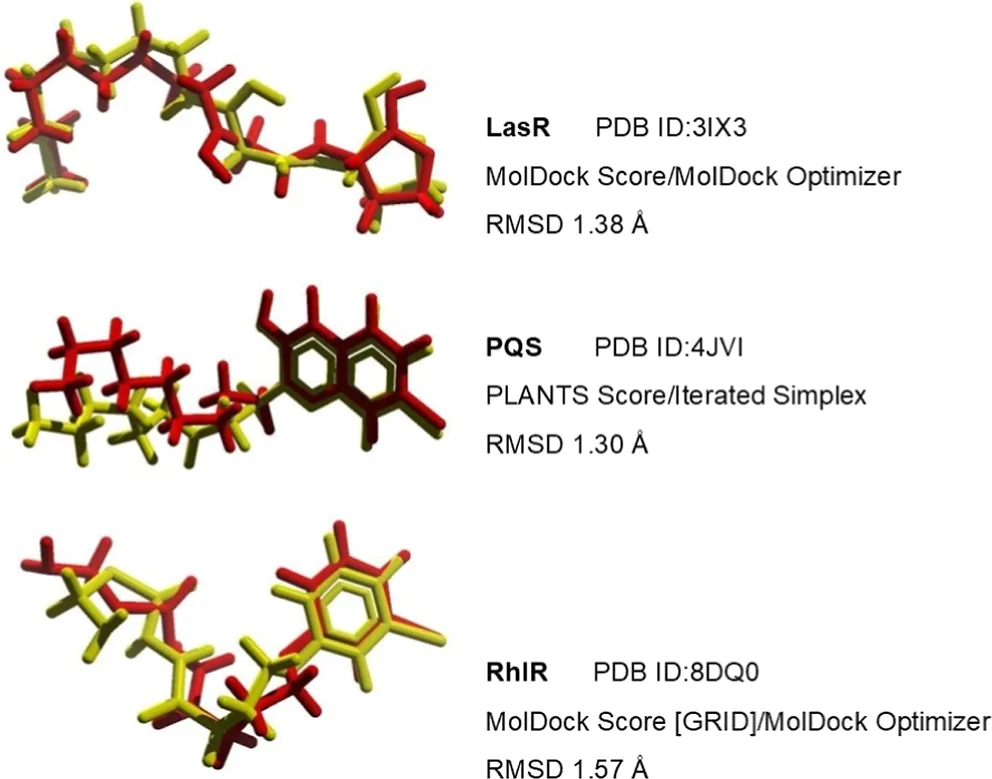
Redocking analysis: the selected methods reproduce the pose of cocrystallized ligands (yellow color) with rmsd less than 2 Å in the superposition of the recoupled ligands (red color). Docking protocols: each method is implemented by a scoring function and a search algorithm: LasR (MolDock Score/MolDock Optimizer), PQS (PLANTS Score/Iterated Simplex), and RhlR (MolDock Score GRID/MolDock Optimizer).

Table 7 shows the affinity energies of curcumin and 5-bromoindole-3-carboxaldehyde on QS proteins, as well as the binding affinity of the endogenous inducers of the proteins LasR (3O–C12–HSL) and RhlR (C4-HSL) and the PQS inhibitor (3NH2–7Cl–C9-QZN).^52^ The amino acids of the ligand-binding domain (LBD) with which the endogenous ligands interact are also shown, as are the two compounds studied as well as the types of ligand–receptor interactions. It should be noted that curcumin is the compound that has the highest affinity energy on the LBD in the 3 proteins, while 5-bromoindole-3-carboxaldehyde is the molecule with the lowest affinity energy; however, it is inferred that it could be a quorum-sensing inhibitor since it binds within the LBDs of the LasR, PQS, and RhlR proteins, as can be seen in Figures 3, 4, and 5.

**Table 7:** Affinity Energy and Binding Mode of Curcumin and 5-Bromoindole-3-carboxaldehyde with *P. aeruginosa* Quorum-Sensing Proteins^a^

target	compounds	Δ*G*kcal/mol	interaction
LasR	3O–C_12_-HSL	–9.29	hydrogen bond (Tyr56, Asp73, Thr75, Ser129), carbon hydrogen bond (Ser29, Tyr93), alkyl (Leu36, Ala50, Val76, Cys79, Ala105, Leu110, Leu125, Ala127), pi-alkyl (Tyr64, Trp88, Phe101).
	curcumin	–11.07	hydrogen bond (Arg61), pi-anion (Asp73), pi-donor hydrogen bond (Tyr56), pi–pi stacked (Tyr64), pi–pi T-shaped (Tyr56), alkyl (Ala105, Leu110), pi-alkyl (Leu36, Trp60, Phe101, Leu110).
	5-bromoindole-3-carboxaldehyde	–7.66	hydrogen bond (Arg61, Tyr93, Leu125), pi-anion (Asp73), pi-donor hydrogen bond (Tyr56), alkyl (Val76, Cys79, Leu125), pi-alkyl (Leu40, Ala50, Trp60, Val76, Phe101, Leu110).
PQS	3NH_2_–7Cl–C9-QZN	–8.23	hydrogen bond (Leu207), alkyl (Ala102, Ile149, Ile186, Ile189, Leu207, Ile236, Pro238, Ile263), pi-alkyl (Ile149, Ala168, Leu207, Leu208, Ile236, Tyr258).
	curcumin	–9.23	hydrogen bond (Gln194, Leu207), carbon hydrogen bond (Ser196, Arg209, Pro210), pi-sigma (Leu208), alkyl (Ile149, Ile236, Pro238), pi-alkyl (Leu208, Phe221, Ile236).
	5-bromoindole-3-carboxaldehyde	–7.14	hydrogen bond (Ile236, Tyr258), carbon hydrogen bond (Ile186, Leu207), pi–pi T-shaped (Tyr258), alkyl (Ile186, Leu189, Leu208, Ile236), pi-alkyl (Ala168, Val170, Leu208, Ile236).
RhlR	C4-HSL	–7.12	hydrogen bond (Try64, Trp68, Asp81, Ser135), alkyl (Ile84, Leu107, Ala111, Val133), pi-alkyl (Trp96, Phe101).
	curcumin	–10.27	hydrogen bond (Trp68), pi-anion (Asp81), pi–pi stacked (Trp96), alkyl (Leu107, Ala111), pi-alkyl (Ala44, Ala83, Phe101, Trp108).
	5-bromoindole-3-carboxaldehyde	–7.68	hydrogen bond (Trp68), carbon hydrogen bond (Pro65, Leu69), pi-donor hydrogen bond (Ser135), pi–pi T-shaped (Tyr72), alkyl (Val60, Leu69, Ile84), pi-alkyl (Ala44, Tyr64, Val60, Ala83).

a The ligand-binding domain of LasR is housed in a large hydrophobic pocket formed by the amino acids Leu36, Gly38, Leu39, Leu40, Tyr47, Glu48, Ala50, Ile52, Tyr56, Trp60, Arg61, Tyr64, Asp65, Gly68, Tyr69, Ala70, Asp73, Pro74, Thr75, Val76, Cys79, Thr80, Trp88, Tyr93, Phe101, Phe102, Ala105, Leu110, Thr115, Leu125, Gly126, Ala127, and Ser129 [49]. Figure 3 shows the interactions between curcumin and 5-bromoindole-3-carboxaldehyde with LBD amino acids and that they share with the endogenous ligand 3O–C12–HSL, such as Tyr56, Asp73, Leu110, and Phe101. In addition, curcumin also interacts with Leu36 and Tyr64 as an endogenous ligand. Meanwhile, 5-bromoindole-3-carboxaldehyde as well as 3O–C12–HSL interact with the residues Ala50, Val76, Cys79, Tyr93, and Leu125.

**Figure 3:**
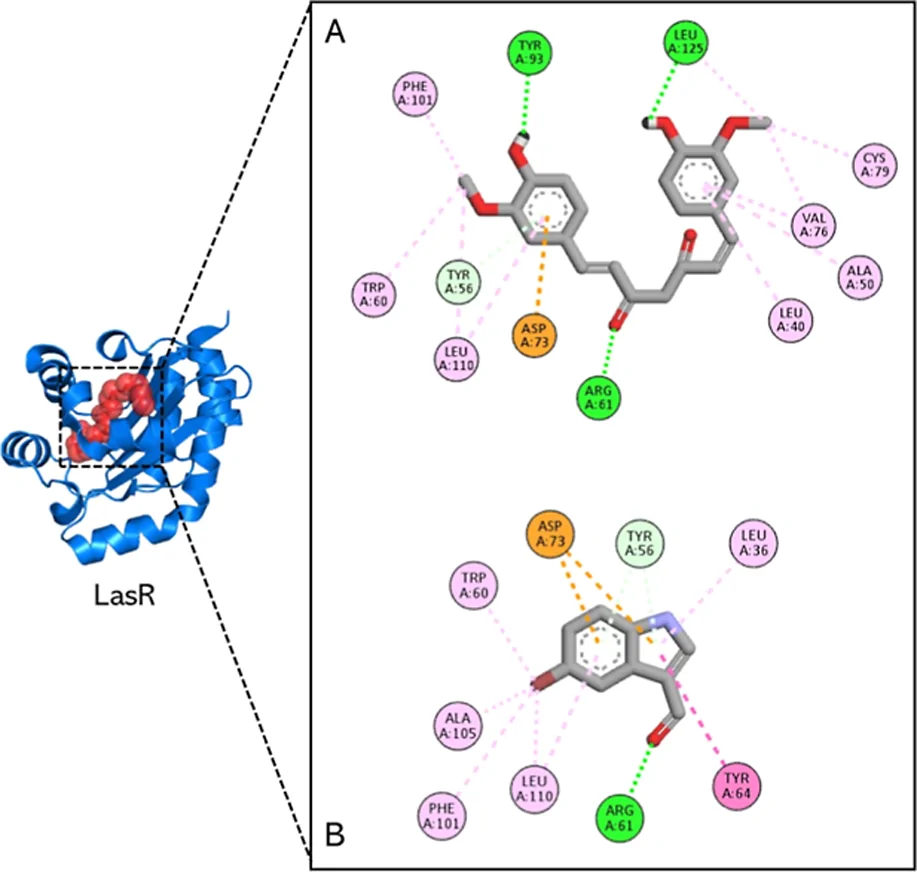
Results of molecular docking on LasR. The image shows the position of the ligand-binding domain (red) on the LasR protein and the 2D interactions of curcumin (A) and 5-bromoindole-3-carboxaldehyde (B).

**Figure 4:**
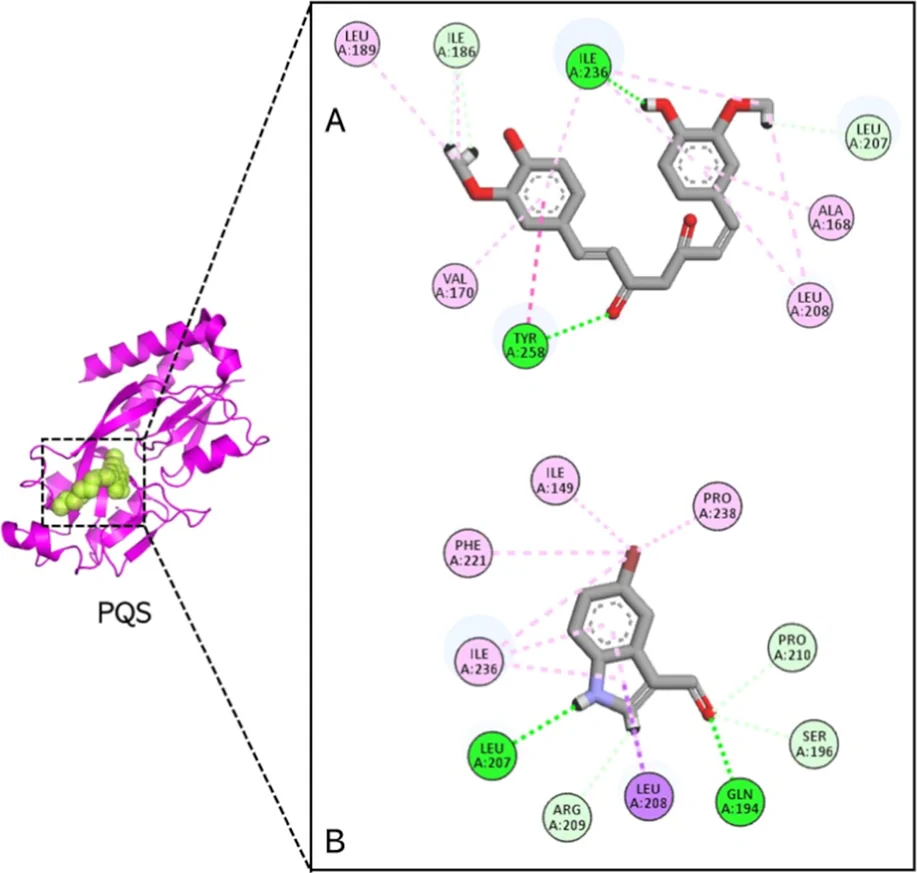
Results of molecular docking on PQS. The image shows the position of the ligand-binding domain (yellow) on the PQS protein and the 2D interactions of curcumin (A) and 5-bromoindole-3-carboxaldehyde (B).

**Figure 5:**
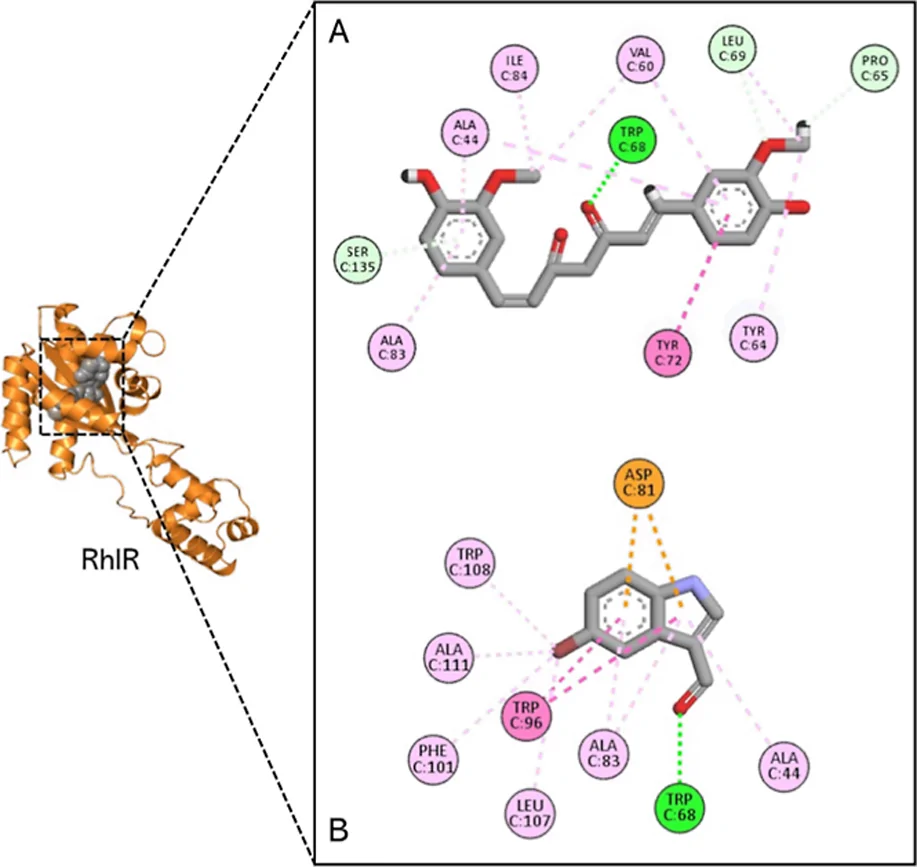
Results of molecular docking on RhlR. The image shows the position of the LBD (gray) on the RhlR protein and the 2D interactions of curcumin (A) and 5-bromoindole-3-carboxaldehyde (B).

Figure 4 shows the structure of the LysR-like transcription factor that responds to the quinolone signal in *Pseudomonas* (PQS) present in *P. aeruginosa*, as well as the ligand-binding domain in yellow that covers residues 91 to 319.^53^ Ilangovan et al., in 2013, evaluated quinazolinone analogues and reported the compound 3NH2–7Cl–C9-QZN (QZN 23) as a novel PQS inhibitor with an IC50 of 5.0 μM,^52^ which binds to the LBD with the amino acids reported in Table 7. The interactions between curcumin and 5-bromoindole-3-carboxaldehyde in the LBD are shown in Figure 4. The QZN-23 inhibitor, curcumin, and 5-bromoindole-3-carboxaldehyde interact with Leu207, Leu208, and Ile236 residues. Curcumin also interacts with the amino acids Ile149 and Pro238 as well as the inhibitor QZN 23, while 5-bromoindole-3-carboxaldehyde like QZN 23 binds with Ala168, Ile186, Ile189, and Tyr258.

It has been reported that for the multimerization and activity of RhlR, its autoinducer C4-HSL is necessary, which binds in the LBD efficiently, and that, in its absence, it homodimerizes, decreasing its capacity as a transcriptional activator.^54^ The residues of the RhlR LBD with which C4-HSL interacts are reported in Table 7. C4-HSL, curcumin, and 5-bromoindole-3-carboxaldehyde bind in LBD and interact commonly with Trp68. Curcumin also binds with other amino acids with which it interacts such as C4-HSL, Asp81, Phe101, Leu107, and Ala111. Meanwhile, 5-bromoindole-3-carboxaldehyde binds with Tyr64, Ile84, and Ser135 residues as well as C4-HSL, as shown in Figure 5.

## Molecular Dynamics Simulations

Since molecular docking does not account for the interactions with the environment surrounding the complexes, the thermodynamic effects, or the intrinsic flexibility of the protein and ligand, we conducted 200 ns MD simulations to further assess the dynamic stability and conformational behavior of the protein–ligand systems. Analyses of hydrogen bonds, root rmsd, RMSF, and an appropriate estimate of the binding energy using the MM/PBSA method allowed us a more comprehensive view of the interactions and validated predictions obtained from molecular docking for complexes involving curcumin and 5-bromoindole-3-carboxaldehyde.

Hydrogen bonds are critical for maintaining the stability of protein–ligand complexes. Figure 6 shows the number of hydrogen bonds formed between curcumin or 5-bromoindole-3-carboxaldehyde and each of the three quorum-sensing proteins over 200 ns of simulation time in MD. The complexes in which curcumin is involved (Figure 6A–C) display a relatively higher and more fluctuating number of hydrogen bonds across the simulation. In particular, the LasR–curcumin suggests a dynamic but consistently engaged interaction network (Figure 6A). The PQS-related protein complex (Figure 6 B) shows a gradual increase in hydrogen-bond formation after approximately 120 ns, which may indicate a progressive stabilization or rearrangement of the ligand within the binding pocket. In contrast, the RhIR–curcumin complex (Figure 6C) exhibits more pronounced fluctuations and intermittent hydrogen bonding, implying a less stable or more transient interaction pattern. For 5-bromoindole-3-carboxaldehyde (Figure 6D–F), the number of hydrogen bonds is generally lower. Notably, the RhIR–5-bromoindole-3-carboxaldehyde complex (Figure 6F) shows the most persistent hydrogen-bonding behavior through simulation time, suggesting a relatively stable binding mode in this specific protein.

**Figure 6:**
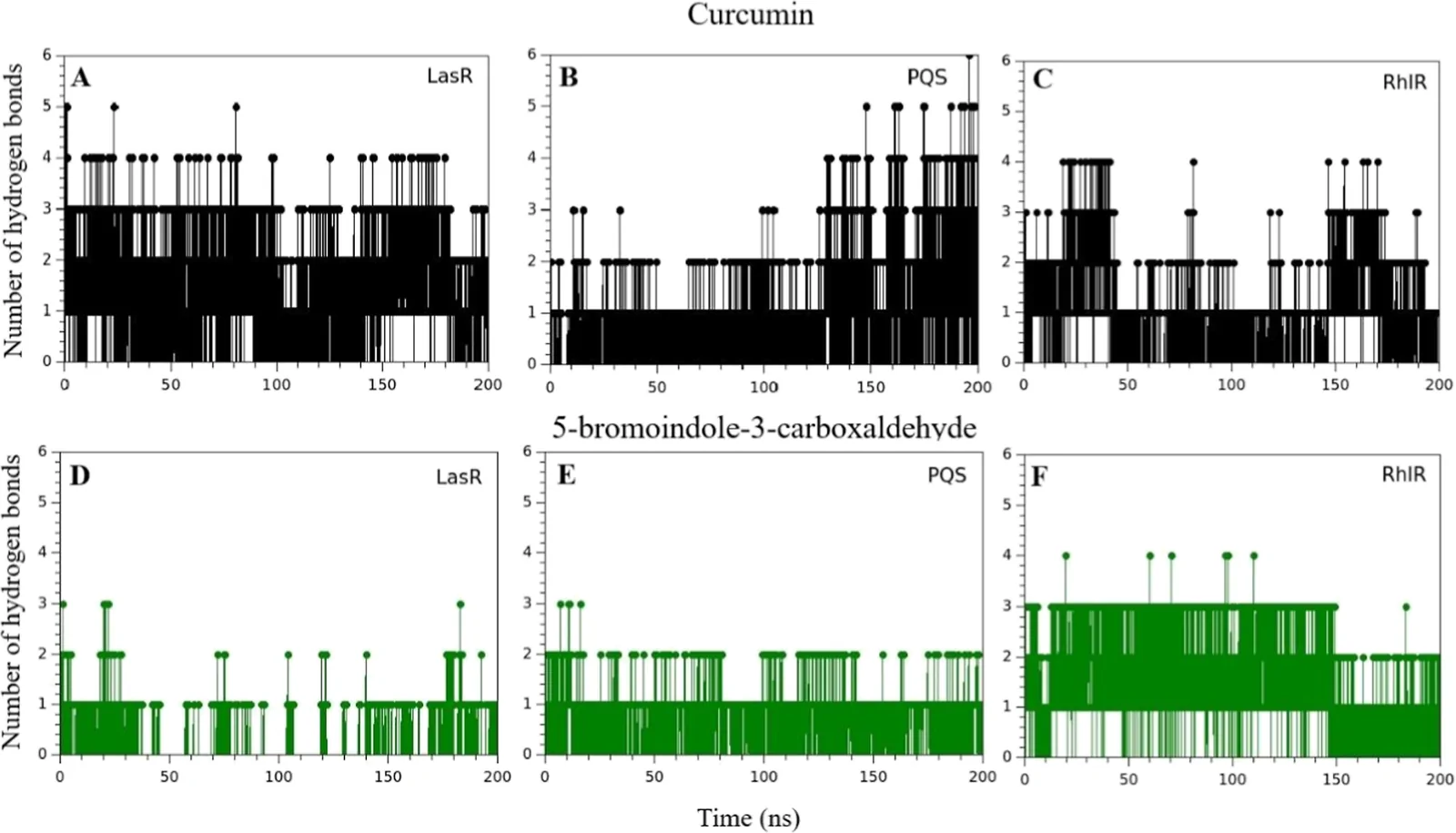
Number of hydrogen bonds as a function of time over 200 ns for the 6 different complexes: (A) LasR-curcumin, (B) PQS-curcumin, (C) RhIR-curcumin, (D) LasR-5-bromoindole-3-carboxaldehyde, (E) PQS-5-bromoindole-3-carboxaldehyde, and (F) RhIR-5-bromoindole-3-carboxaldehyde.

Rmsd was also obtained from MD simulations. Rmsd provides information on the average atomic displacement between two different conformations by least-squares fitting the structure over simulation time to the reference structure, so it is commonly used to assess the structural stability of protein–ligand complexes. Figure 7 presents the rmsd over 200 ns of MD simulation for the protein backbones and the compounds (curcumin and 5-bromoindole-3-carboxaldehyde) in the complexes. For the backbone of each protein, the rmsd in Figure 7 A indicates that LasR and PQS exhibit stable conformations from early times. In contrast, RhIR shows slight fluctuations for the complex with curcumin, stabilizing after ∼100 ns. However, for the complex with 5-bromoindole-3-carboxaldehyde, RhIR displays pronounced rmsd fluctuations, reaching ∼1.2–1.4 nm during the simulation. This rmsd profile suggests enhanced conformational flexibility or partial rearrangement of the protein structure, which tends to stabilize at longer times, indicating that the systems eventually reach dynamic equilibrium. In Figure 7B, the heavy-atom rmsd of curcumin with respect to protein backbone shows a structurally stable conformation for this compound within each of the three proteins throughout the entire simulation time. On the other hand, the heavy-atom rmsd of 5-bromoindole-3-carboxaldehyde with respect to protein backbone shows markedly different behavior depending on the protein. While the ligand remains relatively stable in the PQS and RhlR binding sites, its rmsd in the LasR complex increases significantly, reaching values above ∼6 nm. This large deviation suggests substantial 5-bromoindole-3-carboxaldehyde mobility, possible reorientation, or partial dissociation from the binding pocket, indicating weaker or less stable binding to LasR.

**Figure 7:**
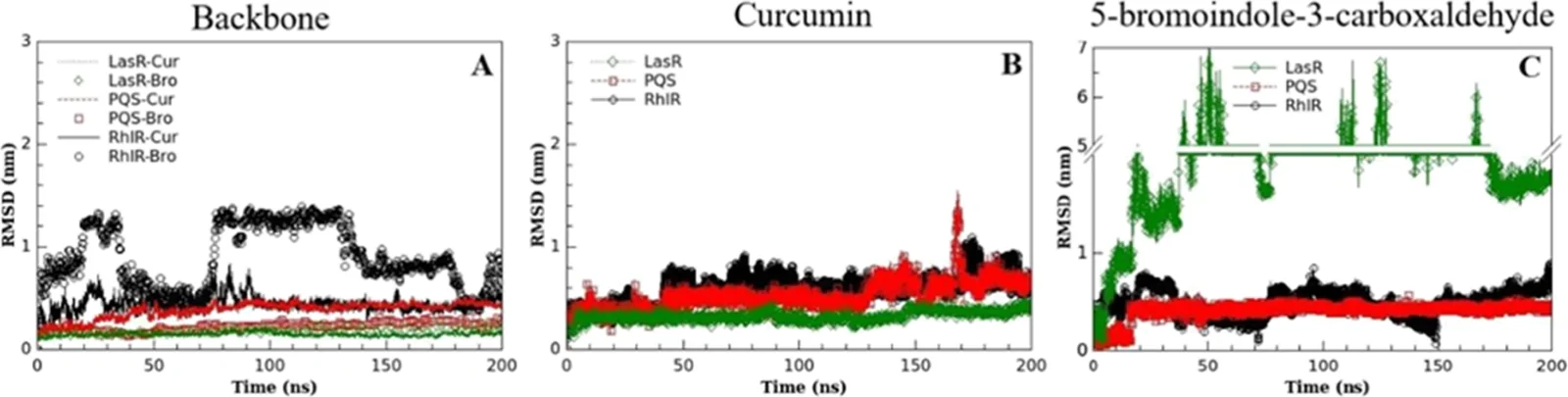
Root mean square deviation (rmsd) over the 200 ns of MD simulations: (A) analysis of protein backbone with respect to the backbone itself (lines or open symbols indicate the presence of curcumin or 5-bromoindole-3-carboxaldehyde, respectively); (B) analysis of heavy atoms of curcumin with respect to protein backbone; and (C) analysis of heavy atoms of 5-bromoindole-3-carboxaldehyde with respect to protein backbone. In all cases, green is for LasR, red for PQS, and black for RhIR.

RMSF complements the structural stability analysis of complexes. RMSF is time-independent but rather the fluctuation of the displacement of an atom relative to its average position over time. For curcumin (left panel of Figure 8), the RMSF analysis shows that, although atomic fluctuations below ∼0.15 nm indicate a certain degree of flexibility, most regions remain nearly rigid across all proteins, suggesting that curcumin is relatively well anchored within the binding sites. Nevertheless, distinct regions of enhanced flexibility are observed, particularly at the terminal aromatic rings. These segments display higher RMSF values, most notably in the RhIR complex. This behavior implies that while the central scaffold of curcumin is stably bound, its terminal groups undergo conformational adjustments that may enable adaptive interactions with the diverse quorum-sensing protein environments. For 5-bromoindole-3-carboxaldehyde, on the right panel of Figure 8, it can be observed that it exhibits substantially lower RMSF across all atoms and protein complexes. Most atoms fluctuate below ∼0.05 nm, indicating a rigid binding mode and limited internal flexibility. A slight increase in RMSF is observed for atoms located close to the aldehyde functional group, particularly in the LasR and PQS complexes, which may reflect localized adjustments to optimize hydrogen bonding or electrostatic interactions.

**Figure 8:**
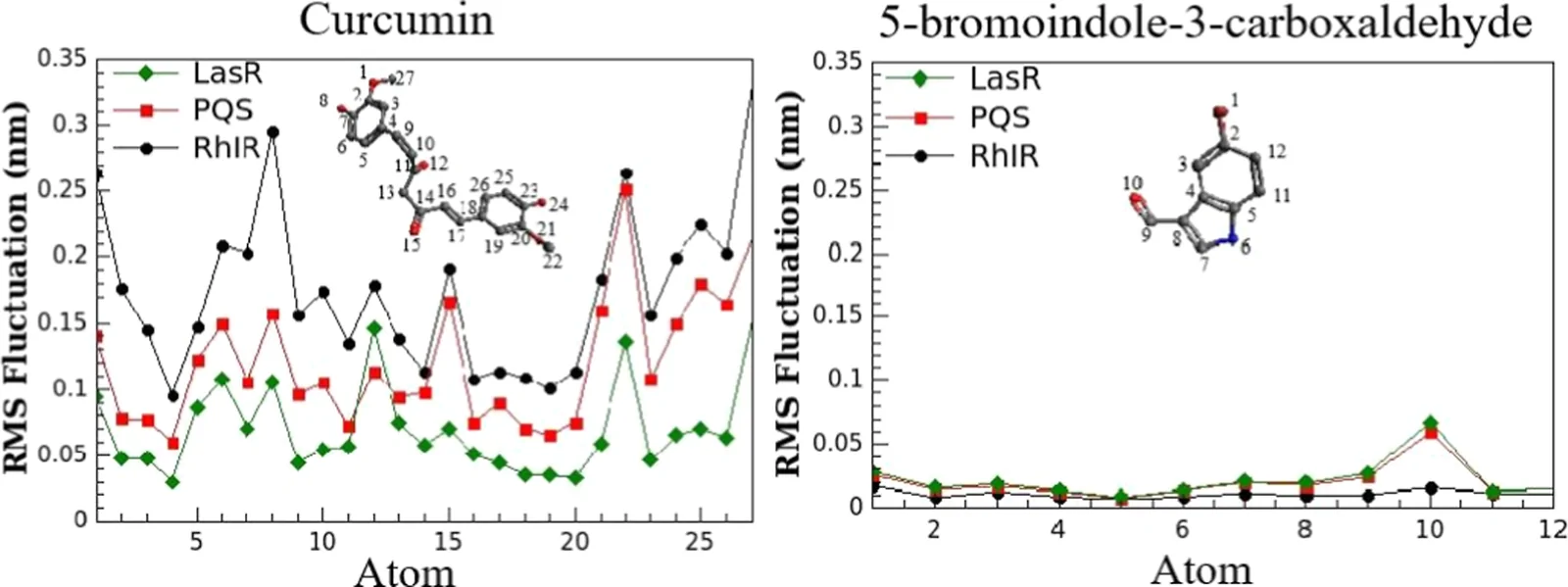
Root mean square fluctuation over 200 ns for the atoms of curcumin (left panel) and 5-bromoindole-3-carboxaldehyde (right panel) when the ligands form complexes with the proteins LasR (green diamonds), PQS (red squares), or RhIR (black circles). The atom number correspondence is according to the structure presented inside each figure.

Figure 9 shows the RMSF of LasR, PQS, and RhIR in the presence of curcumin or 5-bromoindole-3-carboxaldehyde. For LasR (Figure 9A), both complexes show relatively low RMSF, which indicates an overall rigid and stable protein structure during the simulation. In PQS (Figure 9B), moderate fluctuations are observed in several regions, being more evident for curcumin. However, the RMSF remains stable, and no ligand compromises the overall stability of the PQS protein. In the case of RhIR, Figure 9C shows pronounced differences for the two ligands. The complex with 5-bromoindole-3-carboxaldehyde exhibits significantly higher RMSF values, particularly in the C-terminal region, where fluctuations exceeded 1 nm. This indicates a substantial conformational flexibility of the protein.

**Figure 9:**
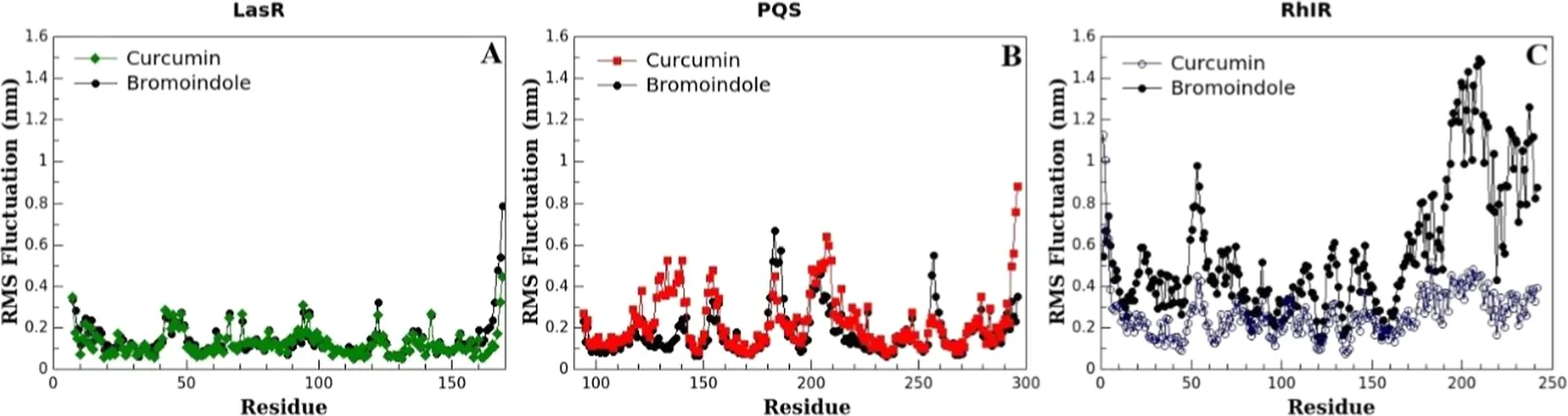
Root mean square fluctuation over 200 ns of the amino acids in proteins: (A) LasR in the presence of curcumin (green diamonds) or 5-bromoindole-3-carboxaldehyde (black circles); (B) PQS in the presence of curcumin (red squares) or 5-bromoindole-3-carboxaldehyde (black circles); and (C) RhIR in the presence of curcumin (blue open circles) or 5-bromoindole-3-carboxaldehyde (black circles).

To quantify the strength of the interaction in complexes, we extend our study by providing a more accurate estimate of the binding energy by means of the MMPBSA approximation. The Gibbs free energy aims to encompass the results obtained by considering the thermodynamic stability of the complexes. In this manner, Table 8 shows electrostatic, van der Waals, polar, and nonpolar contributions to the total binding free energy. As can be seen, in all complexes, gas-phase free energy dominates the binding. Of these, van der Waals interactions are predominant and responsible for the stability of the compounds in the binding pockets due to the presence of hydrophobic contacts. Although electrostatic interactions are also attractive, their contribution is not as significant, so the stability of the complexes is not dictated entirely by the formation of hydrogen bonds. For all systems, polar interactions significantly reduce the affinity between proteins and ligands, reflecting the energy penalty associated with loss of favorable protein/ligand–solvent interactions upon binding. Overall, the binding energy calculations indicate that all protein–ligand complexes are thermodynamically favorable and stable. However, complexes with 5-bromoindole-3-carboxaldehyde exhibit weaker van der Waals and electrostatic interactions, except in the case of RhIR, where the electrostatic contribution is more favorable than that observed for curcumin. Although the polar contribution is lower than curcumin, reflecting reduced desolvation of polar groups, this effect does not compensate for the significantly weaker intermolecular interactions. Notably, the results demonstrate that curcumin presents stronger and more stable binding, validating it as the compound with the highest affinity in the three *P. aeruginosa* quorum-sensing proteins. Because the interaction energy standard deviation (σ_IE_) is greater than 3.6 kcal/mol, as shown in Table 9, the interaction entropy values are not considered.

**Table 8:** MM/PBSA Binding Free Energy Results Expressed in Units of kcal/mol

complex	binding free energy terms					
target	compound	Δ*E*_vdw_	Δ*E*_ele_	Δ*E*_pol_	Δ*E*_nonpol_	Δ*G*_bind_
LasR	curcumin	–49.43 ± 2.91	–14.90 ± 9.21	49.33 ± 8.93	–4.86 ± 0.14	–19.86 ± 6.38
	5-bromoindole-3-carboxaldehyde	–10.13 ± 7.05	–4.75 ± 7.80	8.75 ± 10.42	–1.55 ± 0.88	–7.67 ± 5.46
PQS	curcumin	–37.88 ± 4.16	–14.02 ± 6.05	31.12 ± 6.21	–4.62 ± 0.25	–25.39 ± 6.01
	5-bromoindole-3-carboxaldehyde	–20.00 ± 1.84	–5.89 ± 4.19	17.12 ± 4.31	–2.48 ± 0.10	–11.26 ± 5.42
RhIR	curcumin	–41.33 ± 4.74	–12.01 ± 5.91	33.87 ± 6.62	–4.58 ± 0.22	–24.05 ± 6.13
	5-bromoindole-3-carboxaldehyde	–21.08 ± 2.67	–16.18 ± 6.32	28.46 ± 3.58	–2.47 ± 0.07	–11.26 ± 6.10

**Table 9:** MM/PBSA Binding Entropy (−*T*Δ*S*) and Interaction Energy Standard Deviation (σ_IE_) in kcal/mol

Target	Compound	–*T*Δ*S*	σ_IE_
LasR	curcumin	23.06 ± 0.05	9.75
	5-bromoindole-3-carboxaldehyde	32.86 ± 0.05	12.27
PQS	curcumin	29.16 ± 4.47	8.08
	5-bromoindole-3-carboxaldehyde	10.87 ± 0.05	3.98
RhIR	curcumin	24.01 ± 1.58	8.18
	5-bromoindole-3-carboxaldehyde	15.69 ± 0.05	5.20

## Discussion

The minimum inhibitory concentrations obtained for curcumin correspond to those reported by Adamczak^21^ for Pa01 and ATCC 27853 and Gunes^55^ for strain ATCC 27853, as well as those described by Ramesh^56^ for Pa01 and ATCC 27853 and Gholami^57^ for Pa01. The concentrations in previous studies ranged from 125 to 2000 μg/mL. This shows that the concentrations evaluated for quorum inhibition correspond to subinhibitory concentrations and are comparable to those already reported.

On the other hand, for 5-bromoindole-3-carboxaldehyde, there are no reported MIC studies in *P. aeruginosa*. Therefore, we started with the highest dose evaluated for quorum inhibition in our work, up to 1000 μg/mL, which also demonstrates that we worked with subinhibitory concentrations. The concentrations of 30, 50, and 1000 μg/mL were chosen because the same carboxaldehyde was studied in *C. violaceum* in a study by Kemp^18^ to evaluate quorum inhibition.

It has been shown that *P. aeruginosa* employs quorum sensing in the regulation of genes encoding extracellular virulence factors. Quorum sensing is an intercellular communication system that allows bacteria to control gene expression in a cell population density-dependent manner.^58^

In *P. aeruginosa*, four fundamental QS pathways have been identified. The first is known as the LasI/LasR cascade, and the second includes the RhlI/RhlR pathway. The third is the Pseudomonas quinolone signal (PQS). The fourth is the AI-based integrated quorum-sensing system.^59^

In particular, QS blockade abrogates the virulence without affecting bacterial growth. Therefore, QS represents an attractive target for antivirulence therapy.^31^

To contextualize our findings with what has been reported in the literature, Table 10 summarizes previous studies on curcumin and bromoindole in terms of both MIC and quorum-sensing inhibition. This comparison highlights the consistency of our experimental design and the main differences with established studies and underscores the novelty of the MIC assessment for 5-bromoindole-3-carboxaldehyde in *P. aeruginosa*.

**Table 10:** Reported MIC and Quorum-Sensing Inhibition in *Pseudomonas aeruginosa*

compound	study (year)	Cepa	key QS targets/virulence factor assessed	main findings	present study: novel contribution
curcumin	Rudrappa et al., 2008 [58]	*Pseudomonas aeruginosa* Pa01	QS, LasR	they show significant activity in the inhibition of pyocyanin, elastase, and protease at concentrations of 1.5 to 5 μg/mL.	Three strains of Pseudomonas aeruginosa are evaluated, along with virulence factors such as pyocyanin, protease, elastase, and alginate. A concentration of less than 1.5, which is the minimum in the aforementioned study, is used. MIC, docking studies, and molecular dynamics are performed.
curcumin	Sethupathy et al., 2016 [59]	*Pseudomonas aeruginosa* Pa01 and 32 clinical strains	QS	a significant inhibition of quorum-dependent factors is described at a concentration of 5 μg/mL.	An additional concentration of 1 μg/mL was evaluated in three strains, at which a similar inhibition to that reported with 5 μg/mL was observed. Additional studies such as MIC, docking, and molecular dynamics were also conducted.
curcumin	Fernandes et al., 2023 [60]	*Pseudomonas aeruginosa* Pa14	QS, LasR, LuxS	at a concentration of 200 μg/mL, inhibition of virulence factors is observed.	significant QS inhibition at subinhibitory doses (1–5 μg/mL) and computational modeling.
curcumin-honey	Jaduan et al., 2015 [62]	*Pseudomonas aeruginosa* Pa01	QS	it shows inhibition of virulence factors at concentrations of 50 μg/mL and 1% honey.	efficacy of curcumin alone at lower doses; inclusion of alginate and molecular dynamics.
curcumin	Adamczak et al., 2020 (53)	*Pseudomonas aeruginosa* Pa01	MIC	describe the minimum inhibitory concentration of curcumin for the ATCC 27853 strain and 6 clinical strains.	it shows the effect of curcumin at 2 concentrations and how it inhibits quorum sensing-dependent virulence factors, such as pyocyanin, elastase, protease, and alginate in 3 strains of Pseudomonas aeruginosa: Pa01, Pa14, and Pa ATCC 27853
curcumin	Packiavathy et al., 2014 [15]	*Pseudomonas aeruginosa* Pa01	MIC, QS	it displays the MIC and evaluates antiquorum activity by measuring biofilm inhibition, swarming, rhamnolipids, and alginate at a concentration of 100 μg/mL	three strains of Pseudomonas aeruginosa are evaluated, along with virulence factors such as pyocyanin, protease, and elastase at two lower concentrations and molecular docking studies.
curcumin, curcumin/azithromycin and gentamicin	Bahari et al., 2017 [69]	*Pseudomonas aeruginosa* Pa01, pME3846, and PAO-JP2	MIC, QS	they describe the MIC of curcumin and the synergy in MIC and QS of curcumin and curcumin in addition to azithromycin and gentamicin; the factors evaluated are biofilm and swarming.	three strains of Pseudomonas aeruginosa are evaluated, along with virulence factors such as pyocyanin, protease, and elastase, and molecular docking studies are reported.
electrospun nanofibers loaded with curcumin	Di Salle et al., 2021 [70]	*Pseudomonas aeruginosa* Pa01	Growth inhibition, QS	they report the effect of electrospun nanofibers loaded with curcumin on bacterial growth and their antibiofilm effect.	three strains of Pseudomonas aeruginosa, along with virulence factors at two lower concentrations of curcumin alone.
gallium–curcumin nanoparticle	Ramesh et al., 2022 [53]	*Pseudomonas aeruginosa* ATCC 27853 y 4 clinical strains	MIC and QS	the growth curve and MIC of curcumin-gallium nanoparticles are reported. The inhibition of biofilm formation in an ATCC reference strain and four clinical strains, as well as the MIC, are described.	virulence factors such as pyocyanin, protease, and elastase at two lower concentrations of curcumin alone, the MIC report, and computational modeling.
hydrogel curcumin-hyaluronic acid	Khaleghi et al., 2023 [71]	*Pseudomonas aeruginosa* Pa01	MIC, QS	the MIC and antibiofilm activity and pyocyanin are reported.	three strains of Pseudomonas aeruginosa are evaluated, along with virulence factors such as protease and elastase.
curcumin-ciprofloxacin	Kart et al., 2021 [72]	*Pseudomonas aeruginosa* Pa01, ATCC 27853	MIC, QS	the effect of curcumin in combination with ciprofloxacin on growth inhibition, biofilm formation, swarming, and pyocyanin is described.	virulence factors such as protease and elastase at two lower concentrations and docking and molecular dynamics studies.
5-bromo indole-3-carboxaldehyde	Kemp et al., 2021 [18]	*C. violaceum*	QS	they describe the effect of different bromoindolecarboxaldehydes on violacein production	three strains of Pseudomonas aeruginosa are evaluated, along with virulence factors such as pyocyanin, protease, and elastase at two lower concentrations and docking and molecular dynamics studies.

In a study conducted by Rudrappa in Pa01, where they evaluated different concentrations of curcumin, at concentrations of 1.5 to 3 μg/mL, they achieved an inhibition of pyocyanin of between 60 and 80% of the production with respect to the control, and at 5 μg/mL, even greater inhibition was achieved. Not only did they achieve an inhibition of pyocyanin, but other factors regulated by the QS systems lasI-lasR and rhlI-rhlR were also studied, where they observed a double decrease in the production of elastase and protease and where they also observed that curcumin directly affected the production of the lactones 3-oxoC12-HSL and C4-HSL. In our study we observed that at 1 μg/mL of curcumin, we obtained an inhibition for Pa01 of 60%, which is similar to that observed for Rudrappa, a value similar to that observed in the ATCC 27853 strain.^60^

Sethupathy and collaborators demonstrated the activity of curcumin at 5 μg/mL on the inhibition of pyocyanin, elastase, protease, alginate, and biofilm in 32 clinical isolates and using Pa01 as a control, finding inhibitions that they considered effective inhibition (50–90%), minimal inhibition (20–49%), no inhibition (0–19%), and induced. Where most of the strains had an effectiveness of between 50 and 90% at said concentration, the factors that showed said inhibition are mainly pyocyanin and elastase. The rest of the strains were maintained with minimal inhibition.^61^ In our study, at a concentration of 1 μg/mL for Pa01, an inhibition of 60.60% was obtained, and for 5 mg, 90.63%; for Pa14, an inhibition of 55.89% was achieved at that concentration, whereas the greatest activity was seen in the ATCC 27853 strain, where 100% pyocyanin inhibition was achieved. In addition, similar results were observed in the rest of the virulence factors.

A published study reported by Fernandes showed that curcumin has inhibitory effects on the LuxS/AI-2 and LasI/LasR QS systems, resulting in a reduction of 33%–77% and 21%, respectively. Significantly, curcumin at a high concentration of 200 μg/mL resulted in a 21% decrease in the generation of 3-oxo-C12-HSL by the *P. aeruginosa* Pa14 strain.^62^

Liu and collaborators monitored pyocyanin production and biofilm formation in *P. aeruginosa* when adding curcumin, and they showed a significant decrease in both virulence factors with the molecule.^63^

Gholami and collaborators studied the activity of curcumin and curcumin with metal complexes to inhibit quorum and found important decreases in pyocyanin and considerable amounts of alginate. Their study shows that curcumin alone reduces pyocyanin production by more than half with 67.5% compared to the control in Pa01 and, to a lesser extent, with metals. On the other hand, the activity in alginate with curcumin alone is lower than that shown in combination with metals, with only 21.3%.^57^

Jaduan tested the effect of curcumin mixed with honey to inhibit quorum in *P. aeruginosa* strain Pa01 with the following results: treatment with curcumin (50 μg/mL) and honey (1%) only showed a weak inhibitory effect on siderophore production, a result similar to that observed in treatment with curcumin alone. Curcumin (50 μg/mL) and honey (1%) alone were not found to significantly reduce the activity of Pa01 metalloproteases elastase and protease. Interestingly, curcumin (50 μg/mL) and honey (1%) alone were ineffective in decreasing alginate production.^64^ This synergy shows lower activity than what we obtained in our study for the virulence factors evaluated.

Curcumin is known to act on the lymphatic system. This signaling system is regulated by the production of an autoinducer lactone that modulates the expression of virulence factors, such as alkaline protease and elastase. Therefore, modifications to this system represent a decrease and, consequently, inhibition of the production of these factors, as shown in the results.^61^ If the expression of transcriptional regulators of the lasR/lasI system is affected, then other systems such as PQS and all virulence factors in *P. aeruginosa* will also be affected.^65^

Several studies have described the antibiofilm and antivirulence activities of indole derivatives against clinically important pathogens such as *P. aeruginosa*.^61^ Halogenation of indoles demonstrates a decrease in IC50 and also greater potency as inhibitors of quorum sensing, as described by Odularu et al.^17^ The antimicrobial activity of some indolecarboxaldehydes has been demonstrated in strains of *Pseudomonas sp.*, but there is no information on quorum inhibition in *P. aeruginosa*.^66^

Among the most studied bacterial species, *C. violaceum* has been shown to affect the production of AHLs with good results at concentrations similar to those studied by us.^18^

Indolecarboxaldehydes such as 5-bromoindole-3-carboxaldehyde and derivatives in *Vibrio fischeri* have also been shown to inhibit the LuxI/LuxR system, thereby quenching its bioluminescence or biofilm inhibition in *Vibrio parahaemolyticus*.^18,67^

In *P. aeruginosa*, the following indole derivatives have been found: indole, 3-indolylacetonitrile, 7-fluoroindole, indole-based *N*-acylated l-homoserine lactones, and 2-aminobenzimidazoles, which can inhibit the production of virulence factors and biofilm formation; however, carboxaldehyde derivatives with this activity are not reported.^68^

The solvent did not affect the production of virulence factors at concentrations of 0.01%, 0.5%, and 1% (v/v), according to the prepared stocks of the molecules, since equal results were obtained in the control and negative control; however, some authors report that DMSO can act on quorum sensing at higher concentrations such as 2% (v/v).

The analyses of variance performed for elastase, protease, and alginate activities in *P. aeruginosa* with curcumin and 5-bromoindole-3-carboxaldehyde revealed significant differences only in protease activity between treatments. These results suggest that while some compounds may influence the activity of certain enzymes, not all treatments have a uniform impact on all of the enzyme activities evaluated.

Interestingly, at the lowest concentrations studied, 5-bromoindole-3-carboxaldehyde appeared to stimulate the production of elastase, protease, and alginate, especially in strain ATCC 27853 and, to a lesser extent, in Pa14. This biphasic response is consistent with a hormetic effect, a dose–response phenomenon characterized by stimulation at low doses and inhibition at high doses. This phenomenon has been frequently observed in well-designed studies and is widely generalizable as independent of the chemical/physical agent, biological model, and measured parameter.^69^ In bacteria, there are reports where subinhibitory concentrations of antimicrobial or antivirulence agents regulate gene expression, alter signaling pathways, and activate stress–response mechanisms that can enhance virulence-associated phenotypes instead of suppressing them.^70,71^

In the context of quorum sensing (QS), incomplete inhibition or stimulation at low concentrations can result in partial modulation of the receptor or compensatory upregulation of QS-regulated genes, particularly in species with highly interconnected regulatory networks, such as *P. aeruginosa*. Since QS in *P. aeruginosa* functions as a multilayered regulatory network involving the Las, Rhl, and PQS systems, mild chemical perturbations can trigger nonlinear responses mediated by feedback loops or differential sensitivity among QS pathways.^72,73^

The differential response observed among the three *P. aeruginosa* strains (Pa01, Pa14, and ATCC 27853) is attributed to the genetic and regulatory variability within this species.

*P. aeruginosa* exhibits significant heterogeneity in quorum-sensing (QS) circuits, including differences in the expression and regulation of key systems such as Las, Rhl, and PQS.^72,73^ QS in this organism is not a linear process but rather a complex “network of networks” in which interconnected regulatory circuits integrate environmental and metabolic signals that control population density-dependent gene expression. This hierarchical, multilayered organization allows for the coordinated regulation of numerous virulence-associated genes and contributes to the pathogen’s adaptability.

In this context, strain-specific differences are particularly relevant. The Pa14 strain is more virulent than the reference strain Pa01, despite sharing a high degree of genomic similarity (∼91.7%). Pa14 has a slightly larger genome (∼6.5 Mb vs ∼6.3 Mb in Pa01) and contains distinctive pathogenicity islands, such as PAPI-1 and PAPI-2, which harbor additional virulence-associated genes that are absent or only partially present in Pa01.^74^ These genomic characteristics contribute to differences in virulence gene expression and regulatory dynamics.

On the other hand, mutations or variations in QS regulators, particularly lasR, alter the QS hierarchy and phenotypic responses among strains.^75^ This regulatory variability, along with differences in global regulatory networks and environmental adaptability, could explain the strain-dependent variability observed in the inhibition of virulence factor production—pyocyanin, elastase, protease, and alginate—in the present study. These findings highlight the importance of evaluating antivirulence compounds in multiple strains of *P. aeruginosa*, as quorum-sensing inhibition is strongly influenced by the specific genetic and regulatory background of each strain.

Some limitations of our work are molecular studies to know exactly which gene is repressed and to what extent it is done; however, this represents an opportunity for the future, especially in the case of bromoindole studied, since so far, we are the first to report this activity in three different strains of *P. aeruginosa*.

Chemoinformatic properties related to ADME processes were predicted by using the online predictors pkCSM and SwissADME (Table 6). Absorption properties indicate that the compounds can be administered orally according to the Lipinski rules. Other predicted properties, such as log *P* data and absorption, highlighting intestinal absorption, which is 87.5% for curcumin and 92.3% for 5-BIC, and the skin permeability of both compounds are relatively similar (−2.755 and −2.425, respectively), in addition to the fact that curcumin could inhibit P-GP-1-In, which would cause the compound not to be expelled from the cell and could promote biological activity. On the other hand, 5-BIC appears to permeate the blood–brain barrier, while curcumin does not; this is to be expected due to the difference in size, polar atoms, and solubility, but it opens the possibility that 5-BIC could be used in the treatment of infections affecting the central nervous system.

*In silico* analysis was used to support the hypothesis that curcumin and 5-bromoindole-3-carboxaldehyde interact with the three major quorum-sensing proteins of *P. aeruginosa* (Table 7 and Figures 3–5). Molecular docking studies were performed to obtain the affinity energy and binding mode of the molecules under study in the LBDs of pharmacological targets.

Molecular docking with the LasR protein showed that curcumin has a stronger binding (−11.07 kcal/mol) compared to its endogenous ligand (−9.29 kcal/mol), while 5-bromoindole-3-carboxaldehyde was weaker in affinity (−7.66 kcal/mol). Similarly, curcumin has a higher energy affinity for the PQS protein (−9.23 kcal/mol) compared to the QZN 23 ligand that has an IC50 of 5.0 μM (−8.23 kcal/mol), which we rely on to infer that curcumin could be a PQS inhibitor, while 5-bromoindole-3-carboxaldehyde has a weaker binding (−7.14 kcal/mol). Finally, with the RhlR protein, curcumin also presented a higher affinity energy (−10.27 kcal/mol) compared to the affinity presented by the endogenous ligand C4-HSL (−7.12 kcal/mol), while 5-bromoindole-3-carboxaldehyde presented an affinity like the endogenous ligand (−7.68 kcal/mol). It is important to mention that the result of the affinity energy is only indicative of a higher probability of a more stable bond. Therefore, the binding mode of the ligands under study should be compared with endogenous ligands or other inhibitors or antagonists of the quorum-sensing proteins.

In this sense, Figures 3, 4, and 5 show the binding modes of curcumin and 5-bromoindole-3-carboxaldehyde in the ligand-binding domains of the LasR, PQS, and RhlR proteins. It is shown that both compounds under study can accommodate at the site of activation of LasR and RhlR, suggesting a possible competition or antagonism with their autoinducers in the LBD, and by interacting with essential residues, they promote a disruption of the QS signaling cascade. Likewise, curcumin and 5-bromoindole-3-carboxaldehyde are accommodated in the same LBD cavity as the QZN 23 inhibitor and bind with amino acids relevant to PQS activity, preventing their interaction with DNA and impeding their transcriptional activity. Taken together, the in silico analysis supports the experimental results showing that both curcumin and 5-bromoindole-3-carboxamide are potential inhibitors of QS.

Overall, the molecular dynamics analyses indicate that curcumin interacts more favorably with the quorum-sensing proteins than does 5-bromoindole-3-carboxaldehyde. Curcumin forms a larger and more dynamic hydrogen-bond network, particularly with LasR and the PQS proteins, which contribute to stronger binding. In contrast, 5-bromoindole-3-carboxaldehyde forms fewer hydrogen bonds and exhibits a more rigid, protein-dependent binding mode with reduced stability, especially in the LasR complex.

These observations are supported by structural analysis based on rmsd and RMSF, which indicate that curcumin maintains stable binding throughout the simulations, reduces protein fluctuations, and increases the dynamic stability of the complexes. Conversely, 5-bromoindole-3-carboxaldehyde induces a higher flexibility in specific regions. Consistently, MM-PBSA calculations reveal that although all complexes are thermodynamically favorable, curcumin exhibits stronger van der Waals and electrostatic interactions, resulting in more favorable binding free energies across the three *P. aeruginosa* quorum-sensing proteins.

Although this study evaluated the inhibitory effects of curcumin and 5-bromoindole-3-carboxaldehyde individually, their potential combined activity represents an interesting avenue for future research. According to the molecular docking results, both compounds appear to interact with quorum-sensing-related proteins through distinct binding modes and, possibly, different sites, suggesting that their simultaneous use could generate additive or synergistic effects. These combined interactions could enhance the disruption of the inherently complex, multilinked regulatory networks of quorum sensing.

The concept of combination therapy targeting quorum sensing has gained relevance as a strategy to improve the antiviral efficacy while simultaneously reducing the likelihood of resistance development. Therefore, future studies should explore the combined effects of these compounds using appropriate experimental approaches to determine whether synergistic interactions can be achieved.

Integrating phenotypic assays with computational approaches provides a robust preliminary framework for identifying potential quorum-sensing inhibitors. This study supports the inhibition of quorum sensing using phenotypic assays complemented by molecular docking and molecular dynamics simulations. However, transcriptomic validation (e.g., qRT-PCR analysis of key quorum-sensing regulatory genes such as lasR, rhlR, or pqsR) was not performed. The proposed mechanism is inferred from indirect evidence, including the attenuation of virulence factors and predicted interactions with quorum-sensing regulatory proteins. Future studies should incorporate gene expression analysis to confirm the modulation of quorum-sensing-related pathways at the transcriptional level and to further elucidate the molecular mechanisms underlying the observed antivirulence effects.

Contrary to the premise that antivirulence therapies are impervious to bacterial adaptation, evidence demonstrates that resistance to quorum-sensing (QS) inhibitors is not only possible but also arises rapidly under selective pressure. Experimental studies have described the emergence of resistance in three generations, driven by mechanisms such as the overexpression of efflux pumps (specifically the MexAB-OprM system), mutations in transcriptional regulators (such as vfr), and structural alterations in key receptors, such as LasR.

From an evolutionary perspective, the stability of quorum quenching as a therapy depends on its metabolic context. While selective pressure is lower than that with antibiotics, QS is essential for nutrient acquisition (through the secretion of proteases and siderophores) and for responding to environmental stressors (oxidative, thermal, and osmotic). In hostile or nutritionally limited environments, typical conditions of clinical infection, QS inhibition drastically compromises bacterial fitness, acting as a catalyst for the selection of mutants capable of evading chemical interference.

Clinical evidence in *P. aeruginosa* isolates confirms that these evasion mechanisms are already present in patients, challenging the notion that these therapies are “resistance-free”. Therefore, the use of QS inhibitors should be considered within a combination therapy approach and not as an infallible monotherapy, recognizing that bacterial communication is a trait subject to the same principles of adaptation that govern resistance to conventional antibiotics.^76–80^

Regarding the cytotoxicity and safety of curcumin use, the literature strongly supports the compound’s safety at the concentrations proposed for quorum-sensing inhibition in this work. Multiple umbrella reviews, meta-analyses, and systematic reviews covering more than 25 years of clinical trials confirm that curcumin has a wide safety margin, and it is classified as a GRAS (Generally Recognized as Safe) compound by the FDA.^81,82^ In humans, for various treatments, including anti-inflammatory, antioxidant, and mental health applications, oral doses ranging from 80 mg to 12,000 mg daily have been shown to be well-tolerated without reported serious adverse events or dose-limiting toxicity, even in prolonged treatments of up to 18 months.^53,55,83^

From a cellular perspective, several viability and genotoxicity studies have determined that curcumin maintains DNA integrity and cell vitality at concentrations as low as 5.0 μM. The cytotoxicity threshold only manifests at levels above 10 μM. Phase I clinical trials have confirmed that plasma levels up to 2.0 μM (achieved by intravenous administration of 300 mg/m^2^) do not compromise erythrocyte morphology or systemic homeostasis. The use of low, controlled doses in this study not only guarantees the absence of cytotoxicity but also falls within a therapeutic range that offers antioxidant and protective benefits, validating the safety of the proposed intervention.^84,85^

Structure–activity relationship studies indicate that these bromoindole-derived compounds can achieve potent biological efficacy at micromolar (μM) concentrations, successfully dissociating from systemic cytotoxicity.

Through cell viability assessments (MTT assay) in normal cell lines such as HEK293, it has been validated that bromoindoles maintain host integrity within the therapeutic ranges necessary to interfere with bacterial communication.^17,86,87^

The incorporation of halogen substituents, specifically in bromoindole derivatives, has proven to be an effective strategy for enhancing quorum quenching activity and biofilm inhibition in pathogens such as *P. aeruginosa*. This functional selectivity allows the pathogen to be “disarmed” without resorting to aggressive bactericidal mechanisms, resulting in a superior safety profile compared to conventional chemotherapeutic agents.

Although the present study demonstrates promising in vitro antivirulence and quorum-sensing inhibitory activity of curcumin and 5-bromoindole-3-carboxaldehyde against different strains of *P. aeruginosa*, in vivo validation remains necessary to determine their therapeutic potential under physiological conditions. Factors such as bioavailability, metabolic stability, tissue distribution, toxicity, host immune interactions, and effective concentrations at infection sites may substantially influence their biological activity in living systems. Therefore, future studies should evaluate both compounds using appropriate in vivo infection models to confirm their efficacy and safety as potential antivirulence agents.

## Conclusion

This study provides strong evidence that both 5-bromoindole-3-carboxaldehyde and curcumin are effective in inhibiting quorum-sensing-dependent virulence factors in *P. aeruginosa* in reference strains such as Pa01, Pa14, and ATCC 27853.

5-Bromoindole-3-carboxaldehyde showed significant inhibition of pyocyanin, elastase, protease, and alginate production, particularly in strain Pa01, which showed the greatest reductions in the levels of these virulence factors. The inhibition observed in strains Pa14 and ATCC 27853, although notable, was lower, suggesting that further studies are needed to elucidate the mechanism.

On the other hand, curcumin is a potent inhibitor of the same virulence factors in all strains studied, with a complete inhibition of pyocyanin production in strain ATCC 27853 at a concentration of 5 μg/mL. However, the efficacy of curcumin is variable between strains, with strain Pa14 showing the lowest overall sensitivity to this compound.

These findings support the hypothesis that inhibition of the quorum-sensing system may be an effective strategy to reduce the virulence of *P. aeruginosa* without affecting its growth. The variability observed in the response of the different strains underlines the importance of considering bacterial heterogeneity when developing therapies based on quorum-sensing inhibition.
